# Research Progress on Mono-ADP-Ribosyltransferases in Human Cell Biology

**DOI:** 10.3389/fcell.2022.864101

**Published:** 2022-05-16

**Authors:** Yujie Gan, Huanhuan Sha, Renrui Zou, Miao Xu, Yuan Zhang, Jifeng Feng, Jianzhong Wu

**Affiliations:** ^1^ Jiangsu Cancer Hospital, Nanjing Medical University Affiliated Cancer Hospital, Jiangsu Institute of Cancer Research, Nanjing, China; ^2^ Nanjing Medical University, Nanjing, China

**Keywords:** ADP-ribosylation, PARP, mono-ADP-ribosyltransferases, NAD^+^, MARylation

## Abstract

ADP-ribosylation is a well-established post-translational modification that is inherently connected to diverse processes, including DNA repair, transcription, and cell signaling. The crucial roles of mono-ADP-ribosyltransferases (mono-ARTs) in biological processes have been identified in recent years by the comprehensive use of genetic engineering, chemical genetics, and proteomics. This review provides an update on current methodological advances in the study of these modifiers. Furthermore, the review provides details on the function of mono ADP-ribosylation. Several mono-ARTs have been implicated in the development of cancer, and this review discusses the role and therapeutic potential of some mono-ARTs in cancer.

## 1 Introduction

### 1.1 ADP-Ribosylation

ADP-ribosylation is a post-translational modification (PTM) process that is intrinsically associated with basal metabolic signaling pathways and has been recently identified as an essential regulator of DNA repair and cancer biology (Cohen and Chang, 2018; Crawford et al., 2018). An ADP-ribosyl reaction occurs whenever single or multiple ADP-ribose (ADPr) units present on the redox cofactor β-nicotinamide adenine dinucleotide (β-NAD^+^) are transferred to a substrate protein and when nicotinamide (Nam) is released (Cohen and Chang, 2018). The previously identified modifications occurred on receptor residues (Asp, Glu, Ser, Tyr, Arg, and Cys) linked by O-, N-, and S-glycoside bonds (Cohen and Chang, 2018). With advancements in the detection technology, ADP-ribosylation is no longer considered only as a protein modification, and these modifications have also been reported to occur onto phosphorylated nucleic acids such as the ends of DNA and RNA (Dölle and Ziegler, 2017; Munnur et al., 2019; Groslambert et al., 2021; Weixler et al., 2021).

In mammals, this biochemical reaction is mainly catalyzed by three families of enzymes: 1) *clostridium* toxin-like ADP-ribosyltransferase (ARTs) (ARTCs) catalyze extracellular ADP-ribosylation, 2) diphtheria toxin-like ARTs (ARTDs) catalyze intracellular ADP-ribosylation, and 3) sirtuins (namely, SIRT4, 6, and 7) catalyze ADP-ribosylation in different intracellular compartments. In this review, we focused on the 17ARTD family members in humans, following a recent consensus that “PARP” should be used as a separate term to describe various ARTD family members (Table 1) (Lüscher et al., 2021).

**TABLE 1:** Summary of PARP family.

ADP-Ribosyl transferase	Poly (ADP-Ribosyl) polymerase	Alternative names (previously)	Main activity (Vyas et al., 2014)	Catalytic motif (Vyas et al., 2014)
ARTD1	PARP1		PARylation (long, branched)	H-Y-E
ARTD2	PARP2		PARylation (long, branched)	H-Y-E
ARTD3	PARP3		MARylation	H-Y-E
ARTD4	PARP4	vPARP	MARylation	H-Y-E
ARTD5	TNKS1	tankyrase 1	PARylation (short)	H-Y-E
ARTD6	TNKS2	tankyrase 2	PARylation (short)	H-Y-E
ARTD7	PARP15	BAL3	MARylation	H-Y-L
ARTD8	PARP14	BAL2	MARylation	H-Y-L
ARTD9	PARP9	BAL1	MARylation	
ARTD10	PARP10		MARylation	H-Y-I
ARTD11	PARP11		MARylation	H-Y-I
ARTD12	PARP12	ZC3HDC1	MARylation	H-Y-I
ARTD13	PARP13	ZC3HAV1, ZAP	inactive	
ARTD14	PARP7	TiPARP	MARylation	H-Y-I
ARTD15	PARP16		MARylation	H-Y-Y
ARTD16	PARP8		MARylation	H-Y-I
ARTD17	PARP6		MARylation	H-Y-I

**FIGURE 1:**
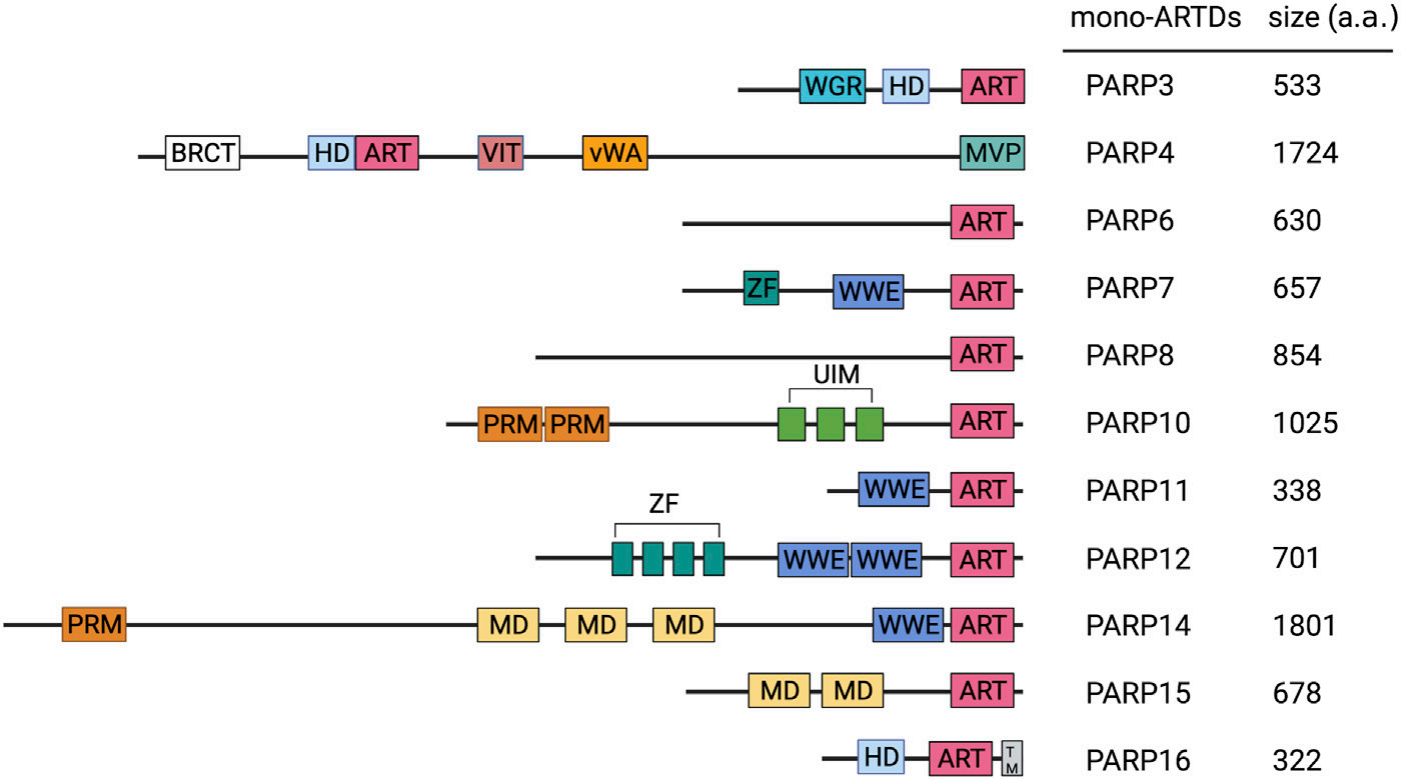
Domain of mammalian MART family members. Important domains of MARTs family members are indicated. ART, ADP-ribosyltransferase domain; BRCT, BRCA1 C terminus domain; HD, helical domain; MD, macrodomain; MVP, major vault protein interaction domain; RRM, RNA-recognition motif; SAM, sterile alpha motif; TM, transmembrane motif; UIM, ubiquitin-interaction motif; vWA, von Willebrand factor type A domain; WGR, conserved Trp-Gly-Arg motif domain; WWE, three conserved residues Trp-Trp-Glu motif domain; ZF, zinc finger motif domain.

Poly (ADP-ribosyl) polymerase (PARP) family share a highly conserved ART folding region, wherein a binding pocket of NAD^+^ is situated, which contains a conserved His-Tyr-Glu (H-Y-E) triplet, also known as an ART signature sequence (Table 1, Figure 1) (Steffen et al., 2013). The sequence differences affect the ability of PARP molecules to transfer ADPr. The first two amino acids (histidine and tyrosine) are critical for NAD^+^ binding, whereas the glutamate residue is essential for elongation of the poly-ADP-ribose (PAR) chain. Glutamate in the catalytic domain of mono-ARTs is replaced by leucine, isoleucine, or tyrosine and restricted to the transfer of one ADPr unit. On the basis of the catalytic efficacy of enzymes, the PARP family can be divided into three groups: poly-ARTs, mono-ARTs, and inactive members. Analysis of self ADP-ribosylation indicates that only PARP1, 2, and Tankyrase (TNKS) 1, 2 can add multiple ADPr units, whereas the remaining 11 PARPs conjugate a single ADPr to amino acid residues (Vyas et al., 2014). Although PARP3, 4 contain H-Y-E patterns, they function as mono-ARTs (Table 1). PARP9, 13 do not have catalytic activity (Vyas et al., 2013). Furthermore, when PARP9 is linked to histone E3 ubiquitin ligase 3L (DT3XL), it can catalyze the action of mono-ARTs (Yang et al., 2017). In particular, the substitutions of His residues in the ART sequence interfere with the binding of NAD^+^, and such substitutions are observed in the catalytically inactive PARP family member, namely, PARP13 (Vyas et al., 2013).

In addition to differences in the catalytic domains, PARP family members also differ in their respective regulation domains. The main functional domains of mono-ARTs are WGR (Trp-Gly-Arg), macrodomains (Macro), WWE (Try-Try-Glu), CCCH Zn Finger, RNA recognition motif (RRM), and ubiquitin-interacting groups (UIM) (Figure 1).

One of the most well-known regions of PARPs is the WGR (Trp-Gly-Arg) domain, which is a conserved central motif essential for DNA-dependent activation (Langelier et al., 2012). Macrodomains, a family of protein domains, play a crucial role in the recognition and combination of ADPr groups (Perina et al., 2014). Multiple macrodomains are present in PARP9, 14, and 15. WWE (Try-Try-Glu) is a domain that has been named after its three most conserved amino acids, and sequence analyses have indicated its association with ADP-ribosylation. PARP7, 12, 13, and 14 have the WWE domain and E3 ubiquitin-protein ligase 1 in common (Barkauskaite et al., 2015). Previously, the WWE domain of RNF146 (ubiquitin E3 ligase) was reported to interact with the PAR chain (Zhang et al., 2011). In addition, the WWE structure of PARP11 binds to the PAR chain; although this interaction may differ from that of RNF146, it may combine with the terminal units of ADPr in the PAR chain. By contrast, PARP14 does not interact with PAR, and evidence indicates that the WWE domain mediates protein–protein interactions independent of protein modifications by PAR (Wang et al., 2012; He et al., 2012).

PARP7, 12, and 13 contain zinc finger domains of the CCCH-type, which are known as RBP binding to RNA (Guo et al., 2004). Zn CCCH is characterized by its ability to bind to both host and viral RNA (Todorova et al., 2014; Guo et al., 2007). Multiple RRMs within PARP10, 14 are involved in RNA binding with high affinity and sequence specificity. In addition, RRMs can interact with ADPr. For example, the RRM domain of the RNA-binding protein, NONO, can bind to PARP1 produced during DNA damage response (Gagné et al., 2012). PARP10 contains two ubiquitin-interacting motifs (UIMs), which can bind to the polyubiquitin chain *via* K63 and can promote interactions between PARP10 and proliferating cell nuclear antigen (PCNA) (Verheugd et al., 2013; Nicolae et al., 2014).

Some mono-ARTs can be classified into multiple groups, whereas other mono-ARTs cannot be classified because they contain domains that are either unique (PARP4, 16) or uncharacterized (PARP6, 8) (Figure 1). The specific functions and significance of these molecules remain further explored, and the development of detection and research tools will hopefully help in discovering other poorly studied mono-ARTs (Steffen et al., 2013).

### 1.2 Factors Affecting ADP-Ribosylation

**FIGURE 2:**
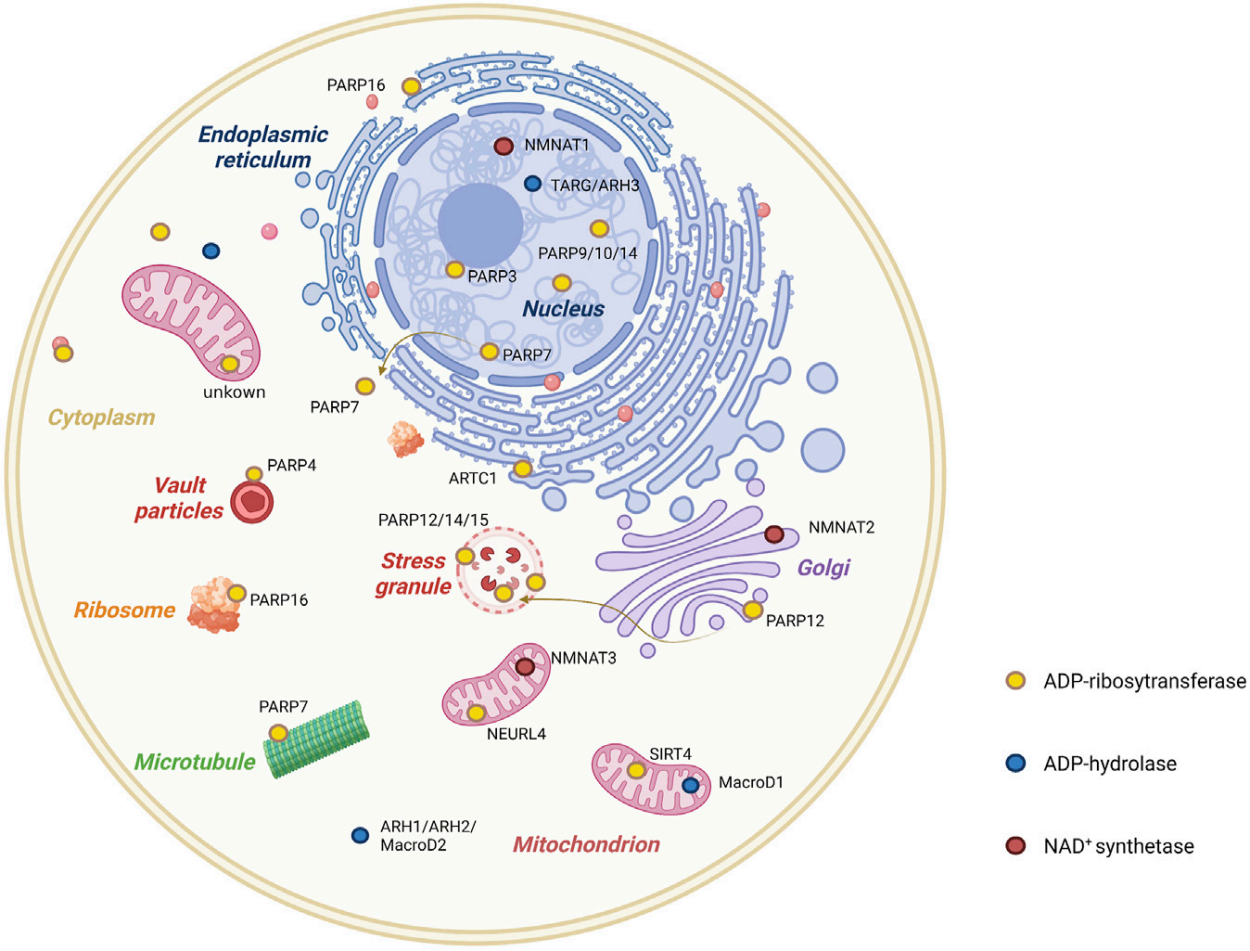
Cellular distribution of enzymes involved in mono-ADP-ribosylation. ADP-ribosylation is a rapidly reversible post-translational modification. Writer (ADP-ribosyltransferases) and eraser (ADP-ribosylhydrases) families are primarily responsible for this pattern, but NAD^+^ concentration in different compartments of the cell also plays a role. As illustrated in the figure, the cellular distribution of mono-ADP-ribosyltransferases, mono-ADP-ribosylhydrases, and NAD^+^ synthases are shown.

ADP-ribosylation is a reversible modification controlled by ARTs (i.e., writers) and removed by the members of two protein families, namely, macrodomains (MacroD) and (ADP-ribosyl) hydrolases (ARHs, i.e., eraser) (Figure 2) (Rosenthal et al., 2013; Cohen and Chang, 2018; Rack et al., 2020).

MacroD1, MacroD2, and terminal ADP-ribose protein glycohydrolase 1 (TARG1) are three macrodomain-containing enzymes capable of reversing the MARylation of proteins and RNA (Munnur et al., 2019; Rosenthal et al., 2013). MacroD1, 2, and TARG1 preferentially remove the ADPr group from acidic residues and hydrolyze a sirtuin by-product, O-acetyl-ADP-ribose (Rosenthal et al., 2013; Chen et al., 2011; Jankevicius et al., 2013). MacroD1 is most prevalent in the mitochondria of skeletal muscles cells. MacroD2 is localized to the nucleolus and cytoplasm and is found only in neuroblastoma cells, whereas the more ubiquitously expressed TARG1 is present in the nucleoplasm, nucleolus, and stress granules (Žaja et al., 2020).

Among ARHs, ARH1 and inactive ARH2 are localized in the cytoplasm, whereas ARH3 is localized in the nucleus, cytosol, and mitochondria (Moss et al., 1992; Niere et al., 2008; Beijer et al., 2021). ARH3 is the main hydrolases of serine-MARylation and consequently plays a critical role in DNA damage response (Rack et al., 2020). In addition, ARH3 can remove the terminal ADP-ribose moiety from the protein substrate, a necessary step for the process of poly-ADP-ribosylation reversal (Rack et al., 2021). On the other hand, poly (ADP-ribose) glycohydrolase (PARG) is incapable of cleaving the terminal ADP-ribosyl bond (Slade et al., 2011), which is responsible for the bulk of polymer degradation.

The concentration of NAD^+^, an ADPr donor, varies across different cellular sub-regions because it does not exhibit transmembrane capability. The NMNAT family includes rate-limiting enzymes of the major NAD^+^ synthesis pathway that are located in different cellular compartments. For example, NMNAT1 is located in the nucleus, NMNAT2 is located in the Golgi membrane, and NMNAT3 is located in the mitochondria (Lau et al., 2009; Ryu et al., 2018). The specific subcellular location of these three NAD^+^ synthases leads to the segregation of NAD^+^ generation, which is critical to maintain NAD^+^ homeostasis within the cell (Figure 2).

## 2 Recent Advances in Methodologies

Although ADP-ribosylation is a fundamental modification, research on this aspect is still in the preliminary stage because of the challenges associated with its detection and identification. In the last 40 years, the development of efficient antibodies and advances in proteomics have facilitated a more thorough understanding of ADP-ribosylation. To date, thousands of ADP-ribosylation modification sites have been identified (Zhang et al., 2013; Bonfiglio et al., 2020).

**FIGURE 3:**
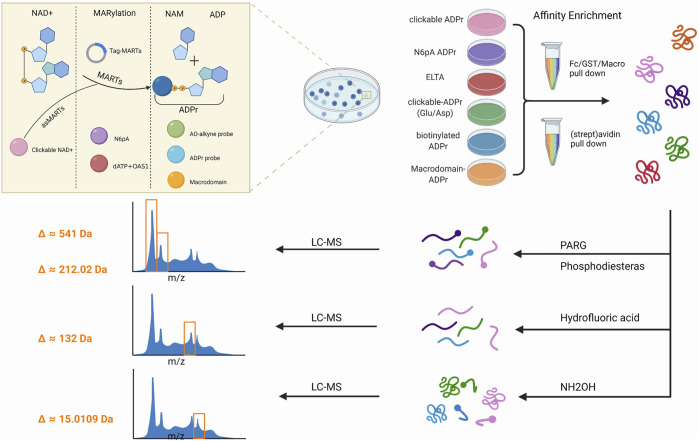
Workflow of mono-ADP-ribosylation (MARylation) detection. The detection of mono-ADP-ribosylated(MARylated) target substrates is divided into three steps. The first step is to use chemical genetics and other methods to label the molecules or products involved in the reaction. A second step involved the use of different affinity systems to enrich the target protein. In the third step, the peptide was treated with an enzyme hydrolysis system before it was subjected to liquid chromatography/mass spectrometry(LC-MS).

The first step in studying this modification is to recognize them Table 2, Figure 3.

**TABLE 2:** Workflow of current strategies for mono-ADP-ribosylation detection.

Step1: Identification		
ADPr detection tools		References
ADPr detection reagent	10H	Kawamitsu et al. (1984)
	ADPr detection reagent	Gibson et al. (2017)
	eAf1521-Fc	Nowak et al. (2020)
	Anti-MAR	Hopp et al. (2021)
	Antibodies for Lys-/Ser-MARylation	Lu et al. (2019); Bonfiglio et al. (2020)
ADPr detection tools	MacroGreen	García-Saura et al. (2021)
	GAP-tag fused toolbox	Sowa et al. (2021)
Labeling the reactants		
Donor	etheno-NAD^+^	
	[32P]-NAD^+^	
	6-biotin-17-NAD^+^	
	NAD^+^ analog	Gibson et al. (2016)
ADP-ribose	AO-alkyne	Morgan and Cohen (2015); Morgan et al. (2017)
	N6-propargyl adenosine (N6pA)	
	Enzymatic labeling of terminal ADP-ribose (ELTA)	Ando et al. (2019)
	Biotinylated ADP-ribose probes	Challa et al. (2021)
Reader	Af1521/eAf1521 macrodomain	Larsen et al. (2017); Nowak et al. (2020)
	PARP14 Macro2/3	Bütepage et al. (2018)
Writer	Tag labeled mono-ARTs	Zhao et al. (2018)
	asPARP	Carter-O'Connell et al. (2016); Gibson and Kraus (2017); Palavalli Parsons et al. (2021)
	BioID	Palavalli Parsons et al. (2021); Carter-O'Connell et al. (2018)
Step2: Enrichment		
Methods	Application	
Affinity pull-down	Dynabeads with Tag or GST	Dani et al. (2009); Zhao et al. (2018)
ADPr-binding domain	Af1521/eAf1521 Macrodomain	Larsen et al. (2017); Nowak et al. (2020)
Strept (avidin) affinity	Dynabeads with streptavidin	Weber et al. (1989); Cho et al. (2020)
Step3: Sample preparation		
Methods	Mass increment	
PARG	+541 Da	Bonfiglio et al. (2020)
Phosphodiesterase	+212 0.02 Da	Daniels et al. (2015)
Hydrofluoric acid	+132 Da	
NH_2_OH reaction	+15.0109 Da	

### 2.1 ADP-Ribose Detection Reagent

In 1984, Kawamitsu et al. developed the first antibody against ADP-ribosylation, named 10H; however, it was thought to bind to only PAR, with a polymer length limit of approximately 10 ADPr units (Kawamitsu et al., 1984). Developing MAR-encoded immunogens that recognize MARylation without responding to PAR and adenosine-derived modifications such as adenylation is challenging.

Researchers have examined naturally occurring ADP-ribosylation-binding domains. The WWE domain recognizes only PARylation, whereas Macrodomain2 and 3 of PARP14 are effective in binding to the MARylation targets (Gibson et al., 2017). In 2017 Gibson et al. synthesized a recombinant antibody-like reagent that recognizes mono-, poly-, and oligo-ADP ribosylations based on the discovery of ADPr binding domains (e.g., Macro and WWE) (Gibson et al., 2017). These reagents can recognize all forms of ADP-ribosylation with different specificities collectively. Moreover, the Macro Af1521 domain of bacteria recognizes both MARylation and PARylation (Larsen et al., 2017). Hottiger Laboratory produced an engineered Af1521-macrodomain that was fused to a mouse Fc fragment (Nowak et al., 2020). After that, using MARylated peptides as antigens, Hottiger Laboratory developed a new commercially available polyclonal ADPr antibody specific for MARylated peptides (Hopp et al., 2021), whose efficacy for detecting ADP-ribosylation was demonstrated in a multi-cancer immunohistochemistry analysis (Aimi et al., 2021). For in-depth research, precisely modified peptides and site-specific antibodies must be developed. Next, an antibody was generated against a peptide with a MARylated lysine in 2019 (Lu et al., 2019). In 2020, Bonfiglio et al. developed a generation of pure Ser-ADP-ribosylated peptides and antibodies capable of detecting site-specific histone Ser-ADP-ribosylation (Bonfiglio et al., 2020). Many reagents are available for studying MARylation; however, none of these reagents has been evaluated by comparing (Table 2).

A recent study compared the currently available reagents, enabling researchers to make an informed decision regarding which reagent to use for their specific applications (Weixler et al., 2022). By using Western blot, Slot blot, and confocal living cell imaging, researchers detected ADP-ribosylation in cells on both protein and RNA substrates. Though no perfect method exists at the moment, future research is expected to focus on developing more efficient tools for detecting the specific ADPr-substrate bond.

### 2.2 Identification Through Reactant Labeling

The construction of ADPr site-specific antibodies requires an in-depth understanding of the modified specific amino acid sites. The ADP-ribosylation reaction is a highly dynamic reversible modification. Furthermore, similar to most PTMs, PARP expression is low in physiological states at the cellular level (Nagaraj et al., 2011), thus requiring the identification and enrichment of mono-ART substrates as a preliminary step before the use of mass spectrometry (MS) analysis. The expression of mono-ARTs is upregulated only under certain conditions such as stress, the presence of interferons, and other cancer-causing factors (Cohen and Chang, 2018). The unstable nature of ADP-ribosylation makes its study challenging. Moreover, it is present in many heterogeneous forms, and its polymers are large and charged.

Combining chemical and genetic engineering methods for the enrichment and identification of ADP-ribosylation sites has been improving over the last decade. A mono-ART substrate detection strategy involves a three-step process that monitors ADP-ribosylation *in vitro* and *in vivo via* various components of the labeling reactants. The following sections discuss the progress made in each category (Table 2, Figure 3).

#### 2.2.1 Donor: Nicotinamide Adenine Dinucleotide

NAD^+^ is the only donor of the PARP family for ADP-ribosylation. Specific protein substrates for PARPs can be identified using labeled NAD^+^ *in vitro* assays. The NAD^+^ analog etheno-NAD^+^ (nicotinamide 1, N6-acetylene dinucleotide) was first synthesized by Barrio et al. (1972). Aubin et al. examined histone H1 ADP-ribosylation using the [32P]-NAD^+^ method in 1982 (Aubin et al., 1982). Bio-NAD^+^ is a 6-biotin-17-nicotinamide-adenine-dinucleotide that was originally developed by Zhang et al. for the detection of MARylated proteins (Zhang and Snyder, 1993). The authors described the biotinylated NAD^+^ synthesis process to produce biotinylated ADP-ribosylated proteins that can be purified using avidin affinity chromatography. However, ARTs may exhibit a lower affinity for labeled NAD^+^ than the natural form, and thus, the targets of most ARTDs are unknown. Moreover, this method cannot evaluate the independent roles of certain ARTD molecules, thus limiting the determination of their specific roles in cellular processes.

For the detection of biomolecules, substrates that contain chemical labels (e.g., azide or alkyne) are excellent chemical reporters for the analysis of protein and nucleic acid modifications. The ability to recognize certain ADP-ribosylated substrate molecules can be achieved using click chemistry to link chemical labels with NAD^+^, a clickable NAD^+^ analog (Gibson et al., 2016). Since the analog has a negligible affinity towards natural PARP, it can only recognize engineered PARPs (e.g., asPARP) (Gibson et al., 2016).

#### 2.2.2 ADP-Ribose

Researchers have developed an aminooxy alkyne probe (AO-alkyne) based on the principle that Glu- and Asp-ADPr bonds can form hydroxylamine derivatives at the modification site after cleavage by hydroxylamine (Morgan and Cohen, 2015). Through cellular labeling, probes can monitor ADP-ribosylation in cells. The MARylated Asp/Glu acid residue can be detected and visualized using the probes by identifying the presence of a free aldehyde. N6-propargyl adenosine (N^6^pA) is administered in intact mammalian cells, and click chemistry is used to generate fluorescently labeled ADPr on the target protein (Westcott et al., 2017). To perform proteomics analysis, N^6^pA-labeled and H_2_O_2_-treated HeLa cell lysates were treated with azido-biotin, purified using streptomycin beads affinity, digested using on-bead protease, and identified using MS. Enzymatic labeling of terminal ADPr (ELTA) is another recently developed method that uses 2′–5′ oligosine synthetase 1 (OAS1) to label 2′-OH analogs of ADPr with dATP, which can be then labeled with fluorescent or affinity labels (Ando et al., 2019). Recently, Kliza et al. used state-of-the-art chemical methods to design and synthesize well-defined biotinylated ADPr probes of discrete lengths (mono-, di-, and tri-ADPr) for affinity purification in conjunction with quantitative MS in mammalian cells to generate ADPr interaction sets of whole proteomes (Kliza et al. 2021).

#### 2.2.3 Reader: ADP-Ribose Binding Domain

The discovery of the ADP-ribose binding domain (ARBD) has provided a new tool for exploring ADPr in cells, and ARBD–GFP fusion allows real-time tracking of the synthesis of local ADPr and PAR in cells (Timinszky et al., 2009). The Macro domain of Af1521, which recognizes MAR and PAR terminal ADPr, can be fused with GST tags to enrich ADP-ribosylation targets in genomics and proteomics screening (Martello et al., 2016). Forst et al. identified the PARP16 macrodomains 2 and 3 as MARylation readers both *in vitro* and in cells and investigated their ability to detect MARylated PARP10 substrates with this approach (Forst et al., 2013). Moreover, the immunoprecipitation of GFP-tagged PARP14 macrodomains supported the hypothesis that PARP16 controls Sec body formation in the absence of amino acids (Aguilera-Gomez et al., 2016). The macrodomain Af1521 is used to establish ADPr-chromatin affinity precipitation and is routinely used for genomic DNA exploration of chromatin-associated proteins for ADPr (Bartolomei et al., 2016).

#### 2.2.4 Writer: Mono (ADP-Ribosyl) Transferase (Mono-ARTs)

Traditional approaches for identifying PARP proteins involved in specific ADP-ribosylation events such as genetic- or RNAi-mediated deletions of specific family members may be inaccurate or produce confounding results. For example, PARP10 can obtain its substrate molecules by constructing Flag and HA label plasmids and co-immunoprecipitation (Zhao et al., 2018); however, this method may cause the omission of substrate proteins to some extent to study specific PARP substrate molecules. Carter-O’Connell et al. developed an NAD^+^ analog-sensitive PARP (asPARP) approach and designed an alkyne NAD^+^ analog to enable Cu-catalyzed click chemistry (Carter-O'Connell et al., 2014). By using the click chemistry method, further visualization of the modification and evaluation of the ability of different ARTDs to modify specific targets is possible. Carter et al. described the use of chemical genetics to label specific targets of a single engineered mono-ART (asPARP) with a clickable NAD^+^ analog containing benzyl substituents at the C-5 position of the nicotinamide ring paired with alkyne groups at the N-6 position of the adenosine ring (5-Bn-6-a-NAD^+^) (Carter-O'Connell et al. 2016). Using this approach, several MARylation specificity targets of PARP10, 11 were identified. Gibson et al. designed a sensitive and clickable NAD^+^ analog, 8-Bu (3-yne) T-NAD^+^ (Gibson and Kraus, 2017), and re-engineered an asPARP developed with the analogous NAD^+^ more efficiently and specifically (Gibson et al., 2016). On this basis, Gibson et al. identified all targets of PARP1, 2, and 3 in a HeLa nuclear extract (Gibson and Kraus, 2017). These studies highlight the usefulness of the asPARP approach in identifying target sublayers of specific PARP family members, thereby providing the possibility of analyzing specific PARP substrate molecules and rapidly expanding the database of PARP targets by using proteological techniques.

Kliza et al. synthesized biotinylated ADPr probes and used them as affinity purification reagents to identify MARylation and PARylation readers in the proteome range, thus resulting in a complete analysis of ADPr proteome and ADPr interactions (Kliza et al., 2021). The aforementioned strategies rely on exogenous biotin, whereas PARP7 uses a proximity-labeling technique known as BioID to identify its intracellular interactors *via* the endogenous expression of biotin (Rodriguez et al., 2021). Using the BioID approach, a protein of interest is fused with a promiscuous biotin ligase (BirA*) (Roux et al., 2013). When biotin is added to cultured cells, BirA* converts biotin to adenylate-biotin, which reacts with proteins proximal to the fusion protein, allowing the identification of intracellular interactors.

Recently, advances in this technology have been made. TurboID and Split-TurboID are more active than the aforementioned biotin ligase–based proximity-labeling methods such as BioID (Cho et al., 2020), leading to a higher temporal resolution and wider *in vivo* application.

### 2.3 Enrichment for ADP-Ribosylation

In each of the aforementioned methods, affinity purification methods are used to induce ADP-ribosylation modification before proteomics analysis and can be classified into the following three types (Dani et al., 2009; Jungmichel et al., 2013): 1. affinity purification *via* labeled vectors, 2. reorganization of ADPr group, and 3. biotin–streptavidin binding system (Table 2, Figure 3).

Because of the dynamic nature and reversibility of ADP-ribosylation, enrichment of ADP-ribosylated substrates by co-immunoprecipitation (Co-IP) would lead to the omission of peptides negatively. Therefore, researchers have used the Macro domain to directly enrich ADP-ribosylation (56). The Af1521 Macro domain has a preference to bind MARylated peptides and a relatively high affinity for ADPr (Kd ∼0.13 μM) (Karras et al., 2005). A study reported that random mutagenesis of wild-type Af1521 led to the development of engineered Af1521 (eAf1521) with a 1000-fold increase in the affinity for ADPr compared with that of the wild-type Af1521 (41). In the proteome ADP-ribosylation MS workflow, its use considerably improved the identification rates of ADP-ribosylation proteins and led to a greater modification coverage.

The biotin and streptavidin systems were developed in the last century. Streptavidin and avidin, collectively known as (strept) avidin, are structurally and functionally similar proteins and have exceptionally high affinity for biotin (Kd ∼10^−14^–10^−16^ M), and their interaction with biotin is much stronger than that of the Macro domain. In addition, they are stable in the presence of heat, denaturants, extreme pH, and proteolytic enzymes (Weber et al., 1989). The “click-it” chemistry technology is used to connect biotin to NAD^+^ analogs or ADPr probes, in addition to strep magnetic beads, for the enrichment of MARylate substrates.

### 2.4 Analysis Strategy of Proteomics

The problem encountered in the identification strategy of proteomics is the similarity between ADPr and other abundant cellular molecules such as adenine nucleotides and nucleic acids. In addition, ADP-ribosylation is catalyzed by various ARTs, each with different enzyme activity and preference for amino acids. Of note, mono-ARTs can modify various amino acid residues (Gibson and Kraus, 2012; Schreiber et al., 2006). To date, ADP-ribosylation has been observed on a wide range of amino acid residues (Glu, Asp, Lys, Arg, His, Cys, and Ser) (Gupte et al., 2017). Currently, the following strategies are available for sample preparation and MS analysis (Table 2, Figure 3).

#### 2.4.1 Detection Strategy for ADP-Ribosylation

In addition to various modified sites, a strong negative charge of the ADPr moiety further makes its identification difficult (Gibson and Kraus, 2012). The MAR and PAR chains have varying lengths, and the differences in their charge complicate the determination of modification levels through proteomics.

Poly (ADP-ribose) glycohydrolase (PARG) is performed *in vitro*, which degradation PAR chains convert into their MARylated counterparts, leaving single ADPr moieties (541 Da) on proteins (Bonfiglio et al., 2020). The similarity between ADPr and other cellular molecules poses several challenges to ADP-ribosylation analysis *via* MS. Furthermore, using the hydrolysis (phosphodiesterase from snake venom) of MAR/PAR into phosphoribose (212.02 Da) can then be analyzed by well-established phosphorylated proteomics methods (Daniels et al., 2014). Snake venom phosphodiesterase I from *Crotalus adamanteus* is available in a partially purified form that requires further purification for its use against ADP-ribosylated proteins (Vyas et al., 2014; Daniels et al., 2014). Alternatively, as lab-friendly tools, NUDIX hydrolases and ectonucleotide pyrophosphatase/phosphodiesterase 1 (ENPP1) also generate phosphoribose acid to generate 5′phosphor-ribose modified proteins *in vitro* (Palazzo et al., 2015; Palazzo et al., 2016; Rack et al., 2020).

Hydrofluoric acid (HF) could be used as a dephosphorylating and phosphodiesterase-like reagent to depolymerise PAR into a unique 132 Da adduct corresponding to the ribose remnant of the ADP-ribose modification (Vyas et al., 2014; Vyas et al., 2013). By combining borate-affinity chromatographic enrichment of ADP-ribosylated peptides with the elution of ADP-ribosylated peptides *via* the NH_2_OH reaction, a hydroxamic acid derivative on glutamate and aspartate residues has a unique mass of 15.0109 Da (Zhang et al., 2013), which can be readily distinguished by MS. Though this approach can identify several sites, a significant limitation is its bias in analyzing aspartate and glutamate residues because other acceptor residues cannot be identified.

The aforementioned strategies have been developed to generate simple derivatives for the effective interrogation of protein databases and site-specific localization of the modified residues. However, only following this way will lead to confusion and make it difficult to differentiate between PARylation and MARylation. Therefore, the comprehensive strategy of binding derivatives and asPARP is a trend in studying PARP-specific protein substrates. The click chemistry combined with genetic engineering can improve identification specificity. Ideally, a method should be applicable to all types of ADP-ribosylation linkages by generating a spectral signature sufficiently simple to be analyzed by general methods developed for LC-MS/MS analysis.

#### 2.4.2 Liquid Chromatography–Mass Spectrometry Strategy

The original PTM remains bound to the analyzed peptide and can be directly detected by MS analysis (Olsen et al., 2010; Choudhary et al., 2009). Contrary to most PTM-based methods, the labile nature of ADP-ribosylation presents a challenge for analyses based on MS with high-energy collisional dissociation (HCD) fragments. After demonstrating the non-energetic fragmentation tendency of electron transfer dissociation (ETD) fragments to include phosphorylation (Molina et al., 2007; Guthals and Bandeira, 2012), ETD with supplemental higher-collisional dissociation (EThcD) has been found to be useful in reliable localization of labile PTMs, including phosphorylation and glycosylation (Frese et al., 2013; Yu et al., 2017). Buch-Larsen et al. recently investigated ADP-ribosylome in its physiological context by combining activated ion ETD (AI-ETD) with unbiased proteomic enrichment of ADPr peptides. AI-ETD identified 120 and 28% more ADPr peptides than ETD and EThcD, respectively (Buch-Larsen et al., 2020). Thus, the authors reported that PARP8 is auto ADP-ribosylated on cysteine residues under physiological conditions. The physiological ADPr of PARP14 targets only tyrosine residues (Buch-Larsen et al., 2020).

The development of improved assays and the enlargement of the database of ADP-ribosylation modifications (ADPriboDB 2.0: http://adpribodb.adpribodb.org) should provide insights into this essential modification.

A combination of biochemistry, genetic engineering, and molecular structure analysis has led to new insights into ADP-ribosylation. However, these methods often require expensive reagents or are unsuitable for large-scale high-throughput screening. Hence, we call for more laboratory-friendly research strategies and detection tools in the future. It is worth noting that two new research tools have recently been reported: MacroGreen and GAP-tag fused molecular toolbox (García-Saura et al., 2021; Sowa et al., 2021).

MacroGreen generated a mutant Af1521 macrodomain fused to the green fluorescent protein (GFP) to generate a high-affinity ADP-ribosyl binding reagent (García-Saura et al., 2021). Staining with MacroGreen allows detection of ADP-ribosylation at sites of DNA damage by fluorescence microscopy. Another technology, the GAP-tag fused molecular toolbox, involves the use of a C-terminal tag based on a Gi protein alpha subunit peptide (GAP), which allows for the site-specific introduction of cysteine-linked mono- and poly-ADP-ribosyl groups or analogs (Sowa et al., 2021). Both tools can be easily produced from *Escherichia coli* and are capable of detecting *in vitro* mono- and poly-ADP-ribosylation of a variety of proteins.

We expect that this broadly applicable tool will facilitate ADP-ribosylation related discoveries, including research studies by laboratories that do not specialize in this field. These methods open ways for the development of various *in vitro* assay systems.

## 3 Location and Function

The aforementioned improved detection methods for ADP-ribosylation have provided new insights into the structure, function, and localization of mono-ARTs. Several studies have demonstrated extensive ADP-ribosylation not only in the nucleus and cytoplasm but also in the subcellular compartments (Nowak et al., 2020; Kliza et al., 2021). Because mono-ARTs are enzymatic in nature, many factors affect the reaction, including substrate concentration, enzyme expression, cellular distribution, and cofactors that regulate its activity (Sanderson and Cohen, 2020). The NAD^+^ concentration varies considerably across different cellular compartments (Cohen, 2020); hence, identifying the location of each mono-ART is crucial for understanding its role within the cell and impact on ADP-ribosylation.

PARP1, 2 is predominantly found in the nucleus, and a recent study by Hottiger using a newly developed anti-ADP-ribose antibody demonstrated heterogeneous ADP-ribosylation staining patterns with predominant cytoplasmic ADP-ribosylation appearance in most cancers (Aimi et al., 2021). Wang et al. detected significant levels of MARylation staining in the cytoplasm of colorectal cancer tissues (Wang et al., 2021). The following sections elaborate on the roles of mono-ARTs based on different subcellular compartments and discuss their impact on carcinogenesis.

### 3.1 Mono-ARTs in the Nucleus

**FIGURE 4:**
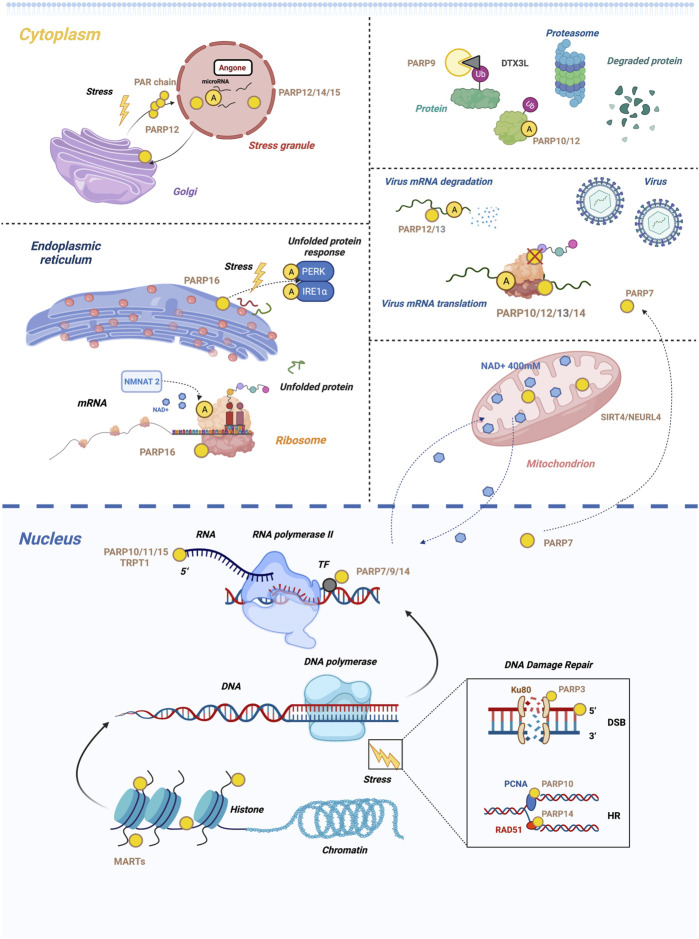
Summary of the biological processes regulated by cellular mono-ARTs. Mono-ARTs are in green, inactive ones are in grey, and blue dots represent NAD^+^. In the nucleus panel, it appears. 1) PARP3 repairs DSBs in response to stress, while PARP10, 14 maintain replication fork stability by another mechanism. 2) PARP7, 9 and 14 are involved in the regulation of gene transcription, and the 5′-phosphorylated end of nucleic acids is also a target of mono-ARTs. In the cytoplasm panel, it appears (described from top to bottom, left to right). 1) Under cellular stress conditions, PARP12 is translocated from the Golgi complex to cytoplasmic SG because it interacts with the PAR generated by PARP1. This is a reversible transposition. 2) PARP-16 regulates UPR by modulating the activity of PERK and IRE1α through MARylation. NMNAT2/PARP16-dependent pathway is involved in the MARylation of ribosomal protein. 3) PARP 9, 10, and 12 are involved in the regulation of ubiquitination. 4) PARP12, 13-mediated RNA degradation of host cell defence. Upon infection with the Sindbis virus (SINV), PARP7 accumulates in the cytoplasm. The dashed line indicates that PARP7 has shifted position. 5) NEURL4 is responsible for ADP-ribosylation in the mitochondria. Mitochondrial ADP-ribosylation impacts nuclear ADP-ribosylation, demonstrating mitochondrial-nuclear NAD^+^ transfer. Dashed lines indicate the NAD^+^ transfer. These functions appear to be controlled by ADP-ribosylation; only the essential functions are presented here, and a detailed description of each can be found in the text.

Because PARP1, 2 are mainly found in the nucleus, PARylation in the nucleus has garnered considerable scientific attention, whereas intranuclear mono-ARTs have been less studied. Recently, with the development of MS techniques and related antibodies and probes, different roles of MARylation in the nucleus have been identified, with PARP1-3, 7, 9, 10, and 14 currently reported to be localized in the nucleus (Table 2, Figure 4) (Sanderson and Cohen, 2020). All PARPs except for PARP1, 2 and TNKS1, 2 are mono ADPr moiety writers. Certain mono-ARTs, such as PARP3, 7, exhibit differential localization in the nucleus and the cytoplasm during different phases of the cell cycle (Vyas et al., 2013).

#### 3.1.1 Role in DNA Damage Repair

##### 3.1.1.1 Recruitment of DNA Repair Factors

Cancer cells are characterized by genomic instability, which is caused by improper or ineffective DNA repair. PARP1, 2, and 3 are known as DNA damage sensors and are rapidly recruited to the DNA damage sites during DNA damage repair (DDR), and they recruit DNA repair factors to facilitate DNA repair. For example, PARP1, 2 are activated after binding to single-strand DNA-binding proteins and promote the recruitment of XRCC1 and ALC1 to the site of injury *via* ADPr of the target protein at the fracture site to regulate the assembly and conversion of other factors promoting DNA repair (El-Khamisy et al., 2003; Ahel et al., 2009; Kim et al., 2015).

In combination with Ku80, PARP3 promotes the repair of double-strand breaks and facilitates the binding of APLF to damaged DNA by driving the classical nonhomologous end-joining pathway (Figure 4) (Fenton et al., 2013; Beck et al., 2014), a therapeutic advantage of PARP3 inhibition that was first demonstrated in 2011. PARP3 deficiency inhibits the growth, survival, and *in vivo* tumorigenicity of BRCA1-deficient triple-negative breast cancer cells or subtypes (Beck et al., 2019).

DNA replication can be interrupted by unrepaired DNA damage or difficult-to-replicate templates as a result of replication arrest (Zafar and Eoff, 2017). To restart stalled replication forks, cells can use two major pathways, namely, homologous recombination (HR) and translesion DNA synthesis (TLS). Once the replication fork is blocked, mono-ubiquitination of PCNA at the Lys164 site promotes the recruitment of TLS polymerase with PIP and UIM of PARP10 to restart replication. Even though PARP10 and PARP14 are structurally and functionally similar (Figure 4), they function differently to cope with DNA damage. PARP14 is a mono-ART necessary for HR but not for TLS, which is responsible for RAD51 recombinase adhesion to damaged DNA (Figure 4). Nicolae et al. reported that RAD51 was MARylated under HR-activity conditions, and PARP14 in S-phase binds to it *via* Macrodomain2 (Nicolae et al., 2015). Using a genome-wide CRISPR knockdown screen, Dhoonmoon et al. reported that PARP14 is an important regulator of the responses to inhibitors of the ATR-CHK1 pathway (Dhoonmoon et al., 2020).

When DNA damage occurs, PARP9, also known as BAL1, and its binding partner BBAP are recruited to the site of damage in a PARP1/PAR-dependent manner *via* the large domains of PARP9 N-terminal macrodomain (Yan et al., 2013). Thus, ubiquitination of histones mediated by BBAP could recruit additional DNA repair factors such as 53BP1 and BRCA1 that are essential for DNA repair (Yan et al., 2013).

##### 3.1.1.2 Histone MARylation

Histone MARylation may also be involved in DNA damage and repair (Karch et al., 2017). Recent studies have shown that ADPr on serine rather than glutamate and aspartate residues in DDR are the dominant and stable forms of the protein ADPr (Bonfiglio et al., 2017). This is made possible by the interaction of PARP1 with histone PARylation factor 1 (HPF1), which changes the tertiary structure of PARP1, 2 granting them specificity for serines (Fontana et al., 2017; Suskiewicz et al., 2020). M. F. Langelier et al. reported that HPF1 binds to PARP1 and PARP2 and inserts a Glu residue to complement the active site; furthermore, there are hydrogen/deuterium exchange mass spectrometry (HXMS) data that support the PARP1/HPF1 interaction in a dynamic manner (Langelier et al., 2021). A study on bacteriocelta showed that H3S10/S28 ADPr is required to inhibit mitotic entry during DNA damage (Brustel et al., 2022). The PARP1/2-HFP1 complexes will redefine the role and significance of mono-ARTs in DNA damage and relevant cell biology. So far, ARH3 is the only known enzyme to reverse serine MARylation, which is responsible for regulating hundreds of ADP-ribosylated proteins in response to DNA damage (Fontana et al., 2017). Rulten et al. reported that MARylation of H1, catalyzed by PARP3, could facilitate double-strand break repair by binding to aprataxin and polynucleotide kinase-like factors (Rulten et al., 2011).

#### 3.1.2 Role in Gene Regulation

Several nuclear PARPs are implicated in gene regulatory outcomes. Current models postulate that PARP regulates gene expression *via* two general mechanisms (Cohen and Chang, 2018): by modulating chromatin structure and (Crawford et al., 2018) by acting as a transcriptional coregulator (Kim et al., 2004; Krishnakumar and Kraus, 2010; Ryu et al., 2015). ADPr molecules have a unique chemical structure that contains two phosphate groups, thus leading to a strong negative charge. ADP-ribosylation of histone or chromatin proteins can induce changes in the spatial structure of chromatin or chromatin remodelers (Boulikas, 1990), all of which can affect chromatin dynamics and alter functions such as DNA repair, transcription, and replication (Stiff et al., 2004).

PARP7 is an essential co-activating transcription factor in the nucleus. A study reported that PARP7 participates in the negative feedback regulation of the aryl hydrocarbon receptor (AHR) signaling pathway (Gomez et al., 2018). AHR is a ligand-activated transcription factor that mediates toxic responses to environmental pollutants such as 2,3,7,8-tetrachlorodibenzo-p-dioxin. It belongs to the basic helix-loop-helix period-AHR nuclear transporter-single-minded (bHLH-PAS) family which regulates immune infections, inflammation, and cancer progression (Stockinger et al., 2014).

PARP7 positively regulates the activity of the liver X receptors (LXRs), LXRα and LXRβ. LXRs, as oxysterol receptors, are important physiological regulators of lipid, cholesterol, and glucose metabolism and inflammatory pathways (Gomez et al., 2018).

Three macro PARPs, namely, PARP9, PARP14, and PARP15, were identified and named as B-aggressive lymphoma (BAL) proteins because of their overexpression in patients with high-risk diffused large B-cell lymphoma and their role as transcriptional regulators (Aguiar et al., 2000; Aguiar et al., 2005). The role of these proteins in the regulation of transcription is discussed in the later section (*Immunity and inflammation*). PARP10 negatively regulates the induction of NF-κ B-dependent genes encoding cytokines. The regulation of NF-κB signaling requires catalytic activity and two unique ubiquitin interaction motifs in PARP10. Ubiquitin interaction motifs recognize K63-linked polyubiquitin and block ubiquitination of the upstream NF-κB activator NEMO, a subunit of the IκB kinase complex (Verheugd et al., 2013).

Overall, ARTs and their modifications play a crucial role in the nucleus; however, the role of mono-ARTs is poorly understood. Understanding the genomic and epigenetic significance of MARylation in carcinogenesis requires more detailed studies and the development of more precise tools.

### 3.2 Mono-ARTs in the Cytoplasm

As mentioned previously, the writers of MARylation are distributed throughout the cell. In 2013, a study by Vyas et al. reported that mono-ARTs are predominantly present in the cytoplasm, and data suggest that PARP8, 12, and 16 potential functions for the assembly or maintenance of membranous organelles (Vyas et al., 2013). Vyas et al. knocked down the PARP family in HeLa cells, and mono-ARTs were discovered to be highly associated with cytoskeletal proteins and their functions, including the regulation of membrane structures, cell viability, cell division, and the actin cytoskeleton (Vyas et al., 2013). Similarly, a study by Kliza et al. reported that MARylation occurs primarily in the dynamic homeostasis of regulatory proteins and that the changes in proteostasis remarkably affect cancer development (Kliza et al., (2021). Protein stability *in vivo* is closely associated with RNA biology and is controlled at many stages, including ribosome biogenesis, ribosomal function, mRNA translation, protein stabilization, protein folding, and removal of misfolded proteins (Kors et al., 2019).

#### 3.2.1 The Role in RNA Biology

##### 3.2.1.1 Ribosomes and mRNAs

Ribosomes are essential components of mRNA translation. The relationship between ribosome generation and ADP-ribosylation was first shown in the crucial role of PARP1 in regulating many steps of ribosome biogenesis, including rDNA transcription, processing, and ribosome assembly in the nucleus (Kim et al., 2019).

A study by Kliza et al. reported that many of the MARylations of cytoplasmic proteins are present on ribosomal proteins (54). However, the studies involving ADP-ribosylation of the ribosomal proteins and their functional implications are still in their developmental stages. An early study by Zhen et al. reported that PARPs modified ribosomal proteins. Interestingly, glutamate- and aspartate-directed ADP-ribosylation was mediated by ribosomal proteins in the breast cancer cell line MDA-MB-468 (Zhen et al., 2017).

MARylation of ribosomes has also been reported in a recent study. Challa et al. reported that the NMNAT2/PARP16-dependent pathway is involved in the MARylation of ribosomal protein (Figure 4) (Challa et al., 2021). MARylation of RPL24 or RPS6 regulates multimer assembly and translation of some mRNAs, which promotes proteostasis and ovarian cancer cell proliferation and is associated with poor clinical outcomes (Challa et al., 2021). Challa et al. also reported the relationship between the MARylation of ribosomal proteins, protein homeostasis, and proliferation of ovarian cancer cells.

##### 3.2.1.2 Stress Granules and mRNAs

Regulation of mRNA translation, stability, and subcellular localization in the cytoplasm is important for the regulation of protein translation during stress. Stress granules (SGs) are nonmembrane-bound organelles formed on stalled ribosomes and are composed of messenger ribonucleoproteins (mRNPs). They maintain and regulate mRNA translation during cellular stress by preventing translation initiation (Wolozin and Ivanov, 2019).

The formation of SG due to cellular stress responses and viral infections is a novel concept which leads to the phosphorylation of eukaryotic initiation factor-2α (eIF2α) (McCormick and Khaperskyy, 2017; Wolozin and Ivanov, 2019). These eIF2α kinases are the key components that integrate stress response, block translation initiation, and facilitate the assembly of SGs. Stress particles reduce the number of available translation factors, thereby inhibiting protein translations.

A study by Leung et al. reported that PARP affects the localization of RBPs to SGs and may contribute to their formation (Leung et al. 2011). Leung found a total of six SG-PARPs, and several of these proteins, including PARP12, 14, and 15, were identified as the components of heat shock-induced stress granules (Figure 4) (Karlberg et al., 2015). Furthermore, X-ray crystallography data showed that PARP12, 13, and 15 were localized at SG (Leung et al., 2006).

Argonaute proteins 1-4 bind small non-coding RNA and are well-known SG components and ADP-ribosylation targets (Karlberg et al., 2015). Argonaute protein levels of ADPr modification are increased due to stress (Leung et al., 2011), which suggests that cytoplasmic mono-ARTs and ADP-ribosylation play a role in the post-transcriptional regulation of gene expression in SGs (Figure 4).

Although the specific role of MARylation in stress granule formation is still unknown, its contribution to the formation of SGs is confirmed. Under cellular stress conditions, PARP12, which is initially localized in the Golgi apparatus, translocates from the Golgi complex to the cytoplasmic SG (Figure 4) (Catara et al., 2017). This process is catalyzed by PARP1 for the formation of PAR, which is released from the nucleus and binds to the WWE domain architecture of PARP12. This leads to the translocation of PARP12 from the Golgi complex to SG. The inhibition of PARP1-mediated PAR formation prevents PARP12 translocation to SG. The translocation of PARP12 to SG is a reversible process; it can be translocated back to the Golgi complex once the stress is relieved (Catara et al., 2017).

A hypothesis based on a recent study suggested that the MARylation hydrolase, nsP3, can inhibit the formation of stress particles (Lu et al., 2021). NsP3 has a conserved N-terminal macrodomain, which hydrolyses mono-ADPr from MARylated protein G3BP1, disassembles virus-induced SG, and inhibits SG formation (Lu et al., 2021). The SGs act as biomolecular condensates. The compartments within cells are formed by a physical process called liquid–liquid phase separation (LLPS) (Protter and Parker, 2016). LLPS is sensitive to changes in the environment and responds to them more rapidly than intracellular transcriptional and translational processes. The regulation of intracellular concentrations of proteins can lead to the formation of high concentrations of localized proteins (Bergeron-Sandoval et al., 2016).

Levels of ADP-ribosylation in the cytoplasm are increased in response to multiple external stimuli (Masutani and Fujimori, 2013). Multiple ADP-ribosylation proteomic studies have suggested an association between the ADP-ribosylation-mediated condensates and diseases such as cancer (Catara et al., 2017). The LLPS hypothesis may provide new insight into the regulation of cellular stress responses by ADP-ribosylation. The development of more precise and effective tools to determine the concentration and structure of ADPr metabolites in cells is needed in the future. Relevant *in vitro* models should be established to elucidate the underlying mechanisms and properties of ADP-ribosylation-mediated agglutination.

#### 3.2.2 The Role Poly-ADP-Riboses in Proteostasis

In addition to affecting mRNA translation, PARPs regulate protein abundance and stability in cells *via* post-translational regulation, which is observed under many stressful conditions (Luo and Kraus, 2012). MARylation mediates proteostasis *via* the following pathways:

##### 3.2.2.1 Protein Degradation Mediated by Ubiquitin

There are pieces of evidence that ADP-ribosylation interacts functionally with ubiquitination, in which the ADPr group functions as a signal for polyubiquitination and the subsequent degradation of the target substrate (Zhang et al., 2011; Kang et al., 2011; Pellegrino and Altmeyer, 2016). Nevertheless, ADP-ribosylation also exhibits antagonistic effects on ubiquitin. Previous studies have reported that bacterial effectors are involved in ADP-ribosylation of ubiquitin to inhibit E1 activation (Qiu et al., 2016; Yan et al., 2020).

PARP 9, 10, and 12 are involved in the regulation of ubiquitination in mono-ARTs (Figure 4). ADP-ribosylation at the C-terminus of ubiquitin is regulated by a complex of PARP9 and Deltex-3-like protein ligase (DTX3L) (Figure 4) (Chatrin et al., 2020). The conserved Deltex RING-DTC domain architecture allows the binding of E2 ubiquitin *via* the RING domain architecture and that of NAD^+^ *via* the DTC domain architecture, which is important for the C-terminal ADP-ribosylation of ubiquitin (Chatrin et al., 2020). PARP10 is exclusive to UIMs, and a study by Verheugd et al. reported that MARylation is a novel PTM that affects NF-KB signaling and prevents the formation of K63-pUb of NEMO (Verheugd et al., 2013). Zhao et al. identified a ubiquitin E3 ligase, RING finger 114 (RNF114), as a novel functional regulator of PARP10 and provided evidence of crosstalk between the components of K27-linked polyubiquitination and MARylation (Zhao et al., 2021). In 2014, Welsby et al. observed that PARP12 enrichment in macrophages is aggregated in structures containing ubiquitinated proteins. A study by Shao et al. reported that PARP12 regulates the stability of the four half-LIM domain architecture proteins FHL-2 (Shao et al., 2018). PARP12, deficiency promoting FHL2 ubiquitination and TGF-β1 expression.

##### 3.2.2.2 Unfolded Protein Response Pathways

Approximately 40% of the proteins in cells are synthesized and folded correctly in the endoplasmic reticulum (ER) (Sun and Brodsky, 2019). Misfolded proteins accumulate in cells, which causes ER stress and activates the unfolded protein response (UPR) (Shacham et al., 2019). ER stress regulates various precancerous characteristics; therefore, ER stress receptors and downstream signaling pathways are the key regulators of tumor growth and metastatic progression and response to chemotherapy, targeted therapies, and immunotherapy.

PARP16 is a tail-anchored protein with catalytic properties (Karlberg et al., 2012). It is localized in the ER and plays an instrumental role in ADP-ribosylation of the ER (Di Paola et al., 2012). The PARP16 protein and its catalytic activity regulate the UPR signaling pathways, such as PERK and IRE1α (Figure 4), which increase their kinase and endonuclease activities, respectively, and are essential for the ER stress response (Jwa and Chang, 2012). Among the three major ER stress sensors, PARP16 activates P-ERK and IRE1α, whereas the third sensor, ATF6, is not regulated by PARP16 (Cybulsky, 2017). The carboxy-terminal tubular tail of PARP16 is also required for its function during ER stress, even though the reason for this is uncertain (Jwa and Chang, 2012). Cells are highly sensitive to ER membrane stress when PARP16 expression is downregulated (Wang et al., 2017), which suggests that PARP16 may be an important inhibitory target for the treatment of cancer, viral infections, and inflammation.

Chaperones in ER facilitate the folding of proteins. Researchers have proposed that GRP78/BiP, the intraluminal chaperone of ER, is a cellular target of human ARTC1 (Figure 2) (Fabrizio et al., 2015). ARTCs are glycosylphosphatidylinositol (GPI)-anchored peripheral enzymes that are secreted or exposed to the extracellular space (Koch-Nolte et al., 2006; Seman et al., 2004). Because these proteins mature in the ER, ARTCs can also play a significant role in MARylation in ER (Stevens and Moss, 2018). Researchers have used ADP-ribosylation staining of Af1521 in cells expressing ARTC1 to show that it co-localizes with GRP78/BiP in the presence of ER, providing strong evidence to support that the modification occurs within the chaperone of ER (Fabrizio et al., 2015).

A chronic state of ER stress and the activation of UPR are the hallmarks of malignant cells (Chen and Cubillos-Ruiz, 2021), which allow cancer cells to adapt to oncogenic and environmental challenges and co-ordinate many immunoregulatory mechanisms to promote malignancy. The ER stress and UPR should be thoroughly studied in the future to rationally design therapeutic interventions that can overcome the present clinical challenges and improve patient outcomes.

#### 3.2.3 Mitochondria and Nicotinamide Adenine Dinucleotide Homeostasis

The NAD^+^/NADH ratio was estimated to be approximately 700–1000 in the nucleus and cytosol and 7–8 in the mitochondria (Williamson et al., 1967; Stubbs et al., 1972; Zhang et al., 2002). Because NAD^+^ is the only known ADPr donor, ADP-ribosylation is strongly associated with the availability and subcellular distribution of NAD^+^ pools. A recent study that monitored NAD + fluxes in diverse cells and organs demonstrated that when DNA damage is induced, cells experience a significant PARP1-dependent loss of NAD+, accounting for around a third of the total NAD^+^ (Liu et al., 2018). The mitochondrial NAD^+^ concentration is high (approximately 400 mM, 40–70% of the cellular NAD^+^ pool) (Di Lisa et al., 2001; Alano et al., 2007). Therefore, the dependence of ADP-ribosylation on NAD^+^ directly results in the modification of mitochondrial biology.

Hopp et al. characterized mitochondrial ADP-ribosylation and its relationship to NAD + homeostasis and determined that there was a negative correlation between mitochondrial function and changes in nuclear ADP-ribosylation, which can be due to NAD + shuttling (Hopp et al., 2021). Hopp et al. propose mitochondrial NAD^+^ is released in order to maintain appropriate nuclear ADP-ribosylation in response to the encountered stress. Because of hyper-activation of PARP1, a high concentration of NAM is produced, which could also be rapidly converted to NMN by NMNAT1, 2. The observation that mitochondrial ADP-ribosylation has an impact on nuclear ADP-ribosylation demonstrates mitochondrial-nuclear NAD^+^ transfer.

Endogenous mitochondrial ADP-ribosylation was visualized using an NAD^+^ analog (3′-azido NAD^+^) through confocal microscopy (Zhang et al., 2019), which showed that this modification is present in the mitochondria and membrane gaps. To date, besides PARP1, SIRT4, ARH3, PARG, and MacroD1 are proposed to be involved in the regulation of ADP-ribosylation in mitochondria (Niere et al., 2008; Hopp and Hottiger, 2021; Ahuja et al., 2007; Niere et al., 2012). NEURL4 is a new member of the ARTD family (named ARTD17) that is responsible for ADP-ribosylation in the mitochondria (Figures 2, 4) (Hottiger et al., 2010; Aravind et al., 2015; Cardamone et al., 2020). In fact, in both human and mouse cells, the loss of NEURL4 results in an almost complete loss of PAR synthesis in the mitochondria. It even contributes to the loss of mitochondrial membrane potential and impaired mitochondrial DNA integrity.

Mass spectrometric identification of mitochondrial ADP-ribosylated proteins helped in identifying six mitochondrial proteins (Hopp et al., 2021). ATP synthase subunits are the predominant mitochondrial targets of ADP-ribosylation, which suggests that ADP-ribosylation mediates the regulation of ATP synthase activity (Hopp et al., 2021). Mitochondrial ADP-ribosylation can affect NAD^+^-dependent processes in other subcellular compartments; therefore, the identification of the writers and erasers involved in mitochondrial ADP-ribosylation turnover is imperative.

Future studies should focus on how cells use the metabolite distribution to control physiological processes due to the dependence of the mitochondrial and nuclear ADP-ribosylation on intracellular NAD^+^ shuttling. The specific role played by mono-ARTs in the mitochondria and overall cancer metabolism under stressful conditions should be investigated.

### 3.3 Immunity and Inflammation

The transcriptional regulation of mono-ARTs by type I and type II interferons (IFNs) indicates their role in immune response and host defence against pathogens (Jiao et al., 2017). At least five human mono-ARTs possessing antiviral activity have been demonstrated on the basis of the following action mechanisms.

The PARP12, 13-mediated RNA degradation pathway is an effective mechanism of host cell defence (Figure 4). The N-terminal regions of PARP12, 13 contain CCCH-type zinc fingers that bind to RNA in a circular conformation which recognizes specific sequences in viral RNA and DNA and degrades retroviral RNA (Bick et al., 2003). PARP10, 12, 13, and 14 are all induced by IFNs and inhibit viral replication (Figure 4) (Atasheva et al., 2012).

PARP7 can bind to TANK-binding kinase 1, a major kinase involved at the beginning of the pathogen-associated molecular pattern pathway, which results in transcription of type I IFN genes. IFN-type I binds to the IFN-α/β receptor and signals *via* the Janus kinase signal transducer and activator of transcription (JAK/STAT) pathway to induce the expression of hundreds of IFN-stimulated genes, which regulate cellular functions upon the recognition of nucleic acids. The genomic instability of cancer cells can lead to the accumulation of aberrant cytosolic nucleic acids, which, in turn, can activate the pattern recognition receptors (PRRs) (Mackenzie et al., 2017; Paludan et al., 2019). In response to cytosolic nucleic acids accumulated because of pathogens or injury, the PRR pathways, which include cyclic GMP–AMP synthase-stimulator of IFN genes (cGAS-STING) and retinoic acid-inducible gene I, activate type I IFNs to promote innate immunity (Paludan et al., 2019; Ivashkiv and Donlin, 2014; Barber, 2015; Härtlova et al., 2015). At low levels, inflammatory signaling may facilitate cancer growth, whereas, at high levels, it may trigger cell death or immune recognition (Cheon et al., 2014).

As a negative regulator of nucleic acid sensing, PARP7 expression is upregulated in cancer to downregulate IFN signaling. PARP7 inhibitors can cause tumors to release IFN, resulting in tumor regression and persistent immunity (Gozgit et al., 2021). The combination of PARP inhibitors and programmed cell death protein 1 (PD-1)/PD-1 ligand (PD-L1) can activate antigen-presenting cells, such as dendritic cells, *via* the cGAS-STING pathway and is effective in BRCA1-deficient tumors (Jiao et al., 2017). In IFNγ-stimulated THP-1 cells, proteomics studies found high levels of PARP9 and PARP14, which were increasingly ADP-ribosylated (Higashi et al., 2019). Interestingly, PARP9, 14 exert anti-inflammatory and pro-inflammatory effects on macrophages, respectively, thereby regulating macrophage activation (Iwata et al., 2016). The expression of PARP9 is controlled by the IFNγ-JAK2-STAT1-IFN regulatory factor 1 signaling pathway, which is essential for the survival of cells in diffuse large B-cell lymphoma (DLBCL), where the host inflammatory response is activated (Juszczynski et al., 2006). In non-interleukin (IL)-4-stimulating conditions, PARP14 represses transcription by recruiting histone deacetylase (HDAC) 2 and HDAC3 to the IL-4-responsive promoter. In the presence of IL-4, the catalytic activity of PARP14 releases HDACs from promoters, thus enabling STAT-6 to bind to promoter regions of its target genes and activating STAT6-dependent transcription (Mehrotra et al., 2011).

### 3.4 Beyond the Protein Substrate: The Role in Nucleic Acids

In addition to altering proteins, ADP-ribosylation reversibly targets DNA and RNA. ADP-ribosylation of DNA was first reported in 2001 with Pierisin-1, which was capable of the MARylation of double-stranded DNA at the N2 position of guanine (Takamura-Enya et al., 2001). *In vitro* experiments have indicated that PARP3 catalyzed MARylation at the 5′-phosphate terminal of nicked DNA, which can serve as a substrate for DNA ligases (Figure 4) (Belousova et al., 2018). Furthermore, a recent study showed that the PARP2-HPF1-mediated bridging of two DNA breaks activates the PARP2 PARylation of proteins, and it would be interesting to determine whether the same activation mechanism also applies to ADP-ribosylation of DNA ends (Bilokapic et al., 2020).

DNA is not the only nucleic acid substrate that can be ADP-ribosylated. Because phosphorylated RNA ends are chemically similar to phosphorylated DNA ends, these modifications can also be targeted *in vitro*, thus expanding the range of substrates for ADP-ribosylation (Munnur et al., 2019). The RRM domain of PARP10 potentially contributes to its catalytic activity toward nucleic acids (Jankevicius et al., 2013). A study showed that PARP10, 11, and 15 MARylate single-stranded RNA (ssRNA) at its 5′ end (Todorova et al., 2014). The use of (32P)-labeled NAD^+^ as an ADPr donor indicated that PARP10 ribosylates 5′ and 3′ ends of phosphorylated ssRNA (Chen et al., 2011) (Figure 4).

In addition, homologues of human TRPT1 found in fungi, archaea, and bacteria may form a non-classical structure at the 5′-phosphorylated end of the RNA (22). This non-classical RNA cap may enhance the stability of RNA by protecting its ends from degradation by nucleases. It can also recruit proteins involved in RNA signaling, similarly to the m7GpppN cap of mRNA, which recruits EIF4E to initiate translation. ADP-ribosylation caps are also thought to inhibit translation, and modifications to RNA by TRPT1 and PARP10 have been reported to increase the resistance of oligonucleotide substrates to CIP treatment, which could be related to “RNA capping” (19, 22).

These modifications have not been detected *in vivo* because of technical challenges, although pieces of evidence suggest that ADP-ribosylation is a potential nucleic acid modification. To conclude, ADP-ribosylation of DNA and RNA promotes the beneficial physiological effects of this modification, thereby revealing its novel cellular functions.

## 4. From Bench to Bedside

### 4.1 Mono-ARTs and Carcinogenesis

Based on the principle of synthetic lethality, PARP inhibitors can effectively kill tumors that harbor mutations in BRCA1 or BRCA2 genes (Helleday, 2011). Because of the successful results with the use of PARP inhibitors, researchers have begun to pay attention to the association between PARP family compounds and malignancies.

A recent study by Fabio et al. using a newly developed anti-ADP-ribosylation antibody showed heterogeneous ADP-ribosylation staining patterns with a predominant cytoplasmic ADP-ribosylation appearance in most cancers (Aimi et al., 2021), including breast, ovarian, colon, lung, and prostate. In colorectal cancer, the intensity of cytoplasmic ADP-ribosylation staining correlates with metastatic development. In breast and advanced ovarian cancer, cytoplasmic ADP-ribosylation is related to overall patient survival; however, the association is not significant in prostate or lung cancer (Aimi et al., 2021). The results of IHC imply that cytoplasmic ADP-ribosylation is tumor-specific.

The function and expression of mono-ARTs may differ depending on the type of cancer. For example, PARP3, 9 are overexpressed in different types of human cancers, including BRCA1-associated cancers (Beck et al., 2019) and DLBCL (Aguiar et al., 2000). PARP6 is a new member of the PARP family that plays a dual role in various cancers. Evidence suggests that PARP6 expression in human colorectal cancer is associated with a positive prognosis (Qi et al., 2016). Alternatively, another study indicated that the treatment with PARP6 inhibitors might cause apoptosis in breast cancer cells because PARP6 contributes to the maintenance of centrosome integrity in breast cancer cells *via* MARylation of checkpoint kinase 1 (Wang et al., 2018). PARP7 expression is decreased in cancers such as breast, liver, colorectal, and other types of cancers. A study pointed out that PARP7 favours tumor progression in ovarian cancer (Palavalli Parsons et al., 2021). PARP14 has been shown to be critical for human multiple myeloma cell survival, and PARP14 levels are strongly linked with cancer progression and poor prognosis (Cohen and Chang, 2018). The expression of PARP family molecules in various cancer types has been discussed in detail and summarized in another review (Sha et al., 2021).

Several recent studies have provided an in-depth understanding of the precise molecular mechanisms and the relationship between ADP-ribosylation and malignancy. PARP4, also known as vPARP, along with major vault protein, is involved in cellular transport, cell signaling, immune response, and multidrug resistance (Berger et al., 2009; Siva et al., 2001; Mossink et al., 2003). PARP7 has been implicated in a variety of biological processes, and a subsequent study on PARP7 reported that PARP7 induces microtubule protein MARylation, thereby promoting microtubule instability and possibly regulating the growth and motility of ovarian cancer cells (Palavalli Parsons et al., 2021). In addition, PARP7 functions as a negative feedback regulator for some oncogenic transcription factors, including HIF-1, c-Myc, and estrogen receptor (ER) (Rasmussen et al., 2021). A theme emerging from the literature is that the localization of PARP7 is context-dependent (Figure 4). PARP7 is primarily localized to the nucleus, and infection with the Sindbis virus (SINV) causes PARP7 to accumulate in the cytoplasm (Kozaki et al., 2017).

PLK1 phosphorylates PARP10 and inhibits PARP10-mediated ubiquitination of NEMO, consequently increasing the activity of NF-κB transcription (Tian et al., 2020). In contrast, MARylation of polo-like kinase 1 (PLK1) inhibits the kinase activity and oncogenic function of PLK1 in hepatocellular carcinoma (HCC) (Tian et al., 2020). PARP10 mono-ADP-ribosylates Aurora A and inhibits its kinase activity, thereby playing an essential role in tumor proliferation and metastasis suppression (Zhao et al., 2018). PARP14 maintains low PKM2 activity in HCC cells by suppressing JNK1, which promotes the Warburg effect and promotes cancer cell proliferation and survival (Iansante et al., 2015). Recently, Challas et al. demonstrated that NMNAT-2 increases the catalytic activity of PARP16, which promotes protein homeostasis in ovarian cancer cells regulating the translation of specific mRNAs to avoid harmful protein aggregation (Challa et al., 2021).

### 4.2 Inhibitors of Mono-ARTs

With a better understanding of the role of mono-ARTs in carcinogenesis and progression, these molecules are gradually emerging as potential targets for cancer treatment. The development of selective inhibitors of mono-ARTs is garnering increasing attention. Presently, selective inhibitors of MARylating PARPs are available for PARP4, 6, 7, 10, 11, 14, and 16, and only one PARP7 inhibitor, RBN-2397 (Gozgit et al., 2021), is currently under phase I clinical trial (ClinicalTrials.gov identifier: NCT04053673).

By screening a library of compounds for the ability to induce mitotic defects, researchers have identified AZ0108 as a potent PARP6 inhibitor, which exerts antitumor effects *in vivo* and induces cell death in breast cancer cells *in vitro* (Wang et al., 2018).

PARP10 is an intriguing target for cancer treatment because it regulates cell proliferation through various processes, including the regulation of ß-catenin and the alleviation of replication and oxidative stress (Schleicher et al., 2018; Wu et al., 2020). Venkannagari et al. established the conditions conducive to the screening of ART inhibitors and identified OUL35, a selective inhibitor of PARP10 (Venkannagari et al., 2016). Murthy et al. modified OUL35 and developed a compound, 4-(benzyloxy) benzamide derivative, which is potent (IC50 = 230 nM) and selective, and like OUL35, it could rescue HeLa cells from PARP10-induced cell death (Murthy et al., 2018). Based on OUL35, Maksimainen et al. developed mono-ART inhibitors by supplementing the TIQ-A scaffold (PARP1 inhibitor) with slight structural changes, which changed the selectivity of the inhibitors from poly-ARTs to mono-ARTs (Maksimainen et al., 2021).

In 2018, Kirby et al. generated a PARP11 selective inhibitor, ITK7, by exploiting structural differences in the active regions of PARPs that facilitate MARylation versus PARylation (Kirby et al., 2018). Recently, Kirby et al. developed a selective PARP4 inhibitor, AEP07, by utilizing structural bioinformatics approaches to target a unique threonine residue (Thr484) in the PARP4 nicotinamide sub-pocket (Kirby et al., 2021).

Owing to the structural similarity of the catalytic domains of the numerous PARP family members, identifying selective PARP inhibitors might be challenging. Thus, addressing other distinctive structural properties of PARPs, such as large domains, may offer a further avenue for developing inhibitors. All of the inhibitors target the PARPs’ catalytic domain, except for the PARP14 inhibitor GeA-69, a kinase inhibitor, which acts as an inhibitor of PARP14 Macrodomain 2 (Moustakim et al., 2018). Potential PARP16 inhibitors decrease the phosphorylation of PERK and IRE1αgenerated by ER stress, eventually promoting cell death (Wang et al., 2017).

A multidisciplinary approach expands the chemical space of mono-ART inhibitors and provides new leads for understanding selectivity in mono-ART inhibition. Wigle et al. developed an active site probe, NanoBRET, which can be used to investigate cellular residence times of PARP inhibitors in live cells (Wigle et al., 2020). The development of more clinically effective and selective mono-ART inhibitors will be helpful in cancer treatment. Of note, these inhibitors may be used in the treatment of non-oncological disorders such as protection against oxidative stress, reduction of inflammatory responses, and neurological diseases (Marcus et al., 2021).

## 5 Conclusion and Prospects

Mono-ARTs represent a class of biologically and therapeutically powerful enzymes that regulate different cellular pathways and play an essential role in cancer. According to Hottiger et al., cytoplasmic ADP-ribosylation levels and patient prognosis vary according to the type of cancer, indicating a differential expression of ARTs in the cytoplasm, consistent with the findings of several recent studies concerning the PARP family and cancer. In another review, we comprehensively discussed the expression of mono-ARTs in different cancer types and the underlying regulatory mechanisms (Sha et al., 2021).

In general, mono-ARTs play an essential role in cell stress response and cancer progression by participating in DNA damage repair, post-transcriptional gene regulation, and mRNA protein homeostasis (Jwa and Chang, 2012; Mehrotra et al., 2011). Furthermore, MARylation exhibits antiviral effects and is involved in specific inflammatory signaling pathways (Iwata et al., 2016; Guo et al., 2019).

Under cellular stress conditions, PARP12, which is localized in the Golgi apparatus, translocates from the Golgi complex to the cytoplasmic SG (Catara et al., 2017). PARP16, a tail-anchored protein, is localized in the ER and plays an instrumental role in the UPR signaling pathways (Di Paola et al., 2012; Karlberg et al., 2012). Challa et al. reported that PARP16 is also involved in the MARylation of ribosomal protein (Challa et al., 2021). NEURL4 is a new member of the ARTD family that is responsible for ADP-ribosylation in the mitochondria. Furthermore, MARylation in mitochondria affects NAD^+^ homeostasis and cellular metabolisms such as oxidative metabolism and lipid metabolism (Hopp et al., 2021). Consequently, future studies should focus on specific mono-ARTs and compartments within cells.

Although the biological functions of some PARPs have been validated, the mechanisms underlying their effects remain unclear. Vyas et al. reported that PARP8 is required for cell viability and localization to the nuclear envelope in HeLa cells; however, the mechanisms underlying these effects remain unknown (Vyas et al., 2013). With the rapid discovery of novel enzymes and functions, this field has become a research hotspot.

Cellular targets of mono-ARTs and their preferred sites for ADPr on the essential substrate should be identified, emphasizing the identification and functional assessment of specific sites of MARylation. In addition, tissue- and cell-type-specific transgenic mouse models will be valuable for understanding the function of mono-ARTs in cancer and exploring new avenues for developing mono-ART inhibitors in the future.

## Glossary

PTM: post-translational modification
mono-ARTs: mono (ADP-ribosyl) transferase
PARP: poly (ADP-ribosyl) polymerase: poly (ADP-ribose) polymerase
ADPr: ADP-ribose
NAD^+^: nicotinamide adenine dinucleotide
NAM: nicotinamide
ART,ADP: ribosyltransferase
TNKS: tankyrases
MARylation: mono-ADP-ribosylation
ARH,ADP: ribosylhydrolases
PARP: poly (ADP-ribosyl) polymerase: poly (ADP-ribose) polymerase
HUWE1: E3 ubiquitin protein ligase 1
UIMs: ubiquitin interacting groups
MS: mass spectrometric
MARylated: mono-ADP-ribosylated
asPARP: engineered PARPs
N^6^pA: N6-propargyl adenosine
OAS1,2′-5′: oligosine synthetase 1
ADPR-CHAP: ADP-ribose/chromatin affinity precipitation
eAf1521: engineering Af1521
SVP: Snake venom phosphodiesterase I
HCD: high-energy collisional dissociation
ETD: electron transfer dissociation
EThcD: ETD with supplemental higher-collisional dissociation
AI-ETD: activated ion ETD
DDR: DNA damage repair
C-NHEJ: classical non-homologous end-joining pathway
TNBC: triple-negative breast cancer
PIP: PCNA-interacting
DSB: double-strand break
dsDNA: double-stranded DNA
ssRNA: single-stranded RNA
RRM: RNA recognition motif
TCDD, 2,3,7,8: tetrachlorodibenzo-p-dioxin
LXR: liver X receptor
BAL: B-aggressive lymphoma
SGs: Stress granules
mRNPs: messenger ribonucleoproteins
PAR: poly-ADP-ribose
LLPS: liquid–liquid phase separation
HCC: hepatocellular carcinoma
DTX3L: Deltex-3-like protein ligase
UPR: unfolded protein response
ER: endoplasmic reticulum
GPI: glycosylphosphatidylinositol
DLBCL: diffuse large B-cell lymphoma
HDACs: histone deacetylases
MVP: major vault protein
TEP-1: telomerase associated protein 1

## References

[B1] Aguiar R. C. T. Takeyama K. He C. Kreinbrink K. Shipp M. A. (2005). B-aggressive Lymphoma Family Proteins Have Unique Domains that Modulate Transcription and Exhibit poly(ADP-Ribose) Polymerase Activity. J. Biol. Chem. 280 (40), 33756–33765. 10.1074/jbc.m505408200 PubMed Abstract | 10.1074/jbc.m505408200 | Google Scholar 16061477

[B2] Aguiar R. C. T. Yakushijin Y. Kharbanda S. Salgia R. Fletcher J. A. Shipp M. A. (2000). BAL Is a Novel Risk-Related Gene in Diffuse Large B-Cell Lymphomas that Enhances Cellular Migration. Blood 96 (13), 4328–4334. 10.1182/blood.v96.13.4328.h8004328_4328_4334 PubMed Abstract | 10.1182/blood.v96.13.4328.h8004328_4328_4334 | Google Scholar 11110709

[B3] Aguilera-Gomez A. van Oorschot M. M. Veenendaal T. Rabouille C. (2016). *In Vivo* vizualisation of Mono-ADP-Ribosylation by dPARP16 upon Amino-Acid Starvation. Elife 5. 10.7554/eLife.21475 PubMed Abstract | 10.7554/eLife.21475 | Google Scholar PMC5127640 27874829

[B4] Ahel D. Hořejší Z. Wiechens N. Polo S. E. Garcia-Wilson E. Ahel I. (2009). Poly(ADP-ribose)-dependent Regulation of DNA Repair by the Chromatin Remodeling Enzyme ALC1. Science 325 (5945), 1240–1243. 10.1126/science.1177321 PubMed Abstract | 10.1126/science.1177321 | Google Scholar 19661379 PMC3443743

[B5] Ahuja N. Schwer B. Carobbio S. Waltregny D. North B. J. Castronovo V. (2007). Regulation of Insulin Secretion by SIRT4, a Mitochondrial ADP-Ribosyltransferase. J. Biol. Chem. 282 (46), 33583–33592. 10.1074/jbc.m705488200 PubMed Abstract | 10.1074/jbc.m705488200 | Google Scholar 17715127

[B6] Aimi F. Moch H. Schraml P. Hottiger M. O. (2021). Cytoplasmic ADP-Ribosylation Levels Correlate with Markers of Patient Outcome in Distinct Human Cancers. Mod. Pathol. 34 (8), 1468–1477. 10.1038/s41379-021-00788-9 PubMed Abstract | 10.1038/s41379-021-00788-9 | Google Scholar 33742140 PMC8295037

[B7] Alano C. C. Tran A. Tao R. Ying W. Karliner J. S. Swanson R. A. (2007). Differences Among Cell Types in NAD+ Compartmentalization: A Comparison of Neurons, Astrocytes, and Cardiac Myocytes. J. Neurosci. Res. 85 (15), 3378–3385. 10.1002/jnr.21479 PubMed Abstract | 10.1002/jnr.21479 | Google Scholar 17853438

[B8] Ando Y. Elkayam E. McPherson R. L. Dasovich M. Cheng S.-J. Voorneveld J. (2019). ELTA: Enzymatic Labeling of Terminal ADP-Ribose. Mol. Cel 73 (4), 845–856. 10.1016/j.molcel.2018.12.022 PubMed Abstract | 10.1016/j.molcel.2018.12.022 | Google Scholar PMC6629254 30712989

[B9] Aravind L. Zhang D. de Souza R. F. Anand S. Iyer L. M. (2015). The Natural History of ADP-Ribosyltransferases and the ADP-Ribosylation System. Curr. Top. Microbiol. Immunol. 384, 3–32. 10.1007/82_2014_414 PubMed Abstract | 10.1007/82_2014_414 | Google Scholar 25027823 PMC6126934

[B10] Atasheva S. Akhrymuk M. Frolova E. I. Frolov I. (2012). New PARP Gene with an Anti-alphavirus Function. J. Virol. 86 (15), 8147–8160. 10.1128/jvi.00733-12 PubMed Abstract | 10.1128/jvi.00733-12 | Google Scholar 22623789 PMC3421642

[B11] Aubin R. J. Dam V. T. Miclette J. Brousseau Y. Huletsky A. Poirier G. G. (1982). Hyper(ADP-ribosyl)ation of Histone H1. Can. J. Biochem. 60 (12), 1085–1094. 10.1139/o82-139 PubMed Abstract | 10.1139/o82-139 | Google Scholar 6299482

[B12] Barber G. N. (2015). STING: Infection, Inflammation and Cancer. Nat. Rev. Immunol. 15 (12), 760–770. 10.1038/nri3921 PubMed Abstract | 10.1038/nri3921 | Google Scholar 26603901 PMC5004891

[B13] Barkauskaite E. Jankevicius G. Ahel I. (2015). Structures and Mechanisms of Enzymes Employed in the Synthesis and Degradation of PARP-dependent Protein ADP-Ribosylation. Mol. Cel 58 (6), 935–946. 10.1016/j.molcel.2015.05.007 PubMed Abstract | 10.1016/j.molcel.2015.05.007 | Google Scholar 26091342

[B14] Barrio J. R. Secrist J. A. 3rd Leonard N. J. (1972). A Fluorescent Analog of Nicotinamide Adenine Dinucleotide. Proc. Natl. Acad. Sci. U.S.A. 69 (8), 2039–2042. 10.1073/pnas.69.8.2039 PubMed Abstract | 10.1073/pnas.69.8.2039 | Google Scholar 4340748 PMC426863

[B15] Bartolomei G. Leutert M. Manzo M. Baubec T. Hottiger M. O. (2016). Analysis of Chromatin ADP-Ribosylation at the Genome-wide Level and at Specific Loci by ADPr-ChAP. Mol. Cel 61 (3), 474–485. 10.1016/j.molcel.2015.12.025 PubMed Abstract | 10.1016/j.molcel.2015.12.025 | Google Scholar 26833088

[B16] Beck C. Boehler C. Guirouilh Barbat J. Bonnet M.-E. Illuzzi G. Ronde P. (2014). PARP3 Affects the Relative Contribution of Homologous Recombination and Nonhomologous End-Joining Pathways. Nucleic Acids Res. 42 (9), 5616–5632. 10.1093/nar/gku174 PubMed Abstract | 10.1093/nar/gku174 | Google Scholar 24598253 PMC4027158

[B17] Beck C. Rodriguez-Vargas J. M. Boehler C. Robert I. Heyer V. Hanini N. (2019). PARP3, a New Therapeutic Target to Alter Rictor/mTORC2 Signaling and Tumor Progression in BRCA1-Associated Cancers. Cell Death Differ 26 (9), 1615–1630. 10.1038/s41418-018-0233-1 PubMed Abstract | 10.1038/s41418-018-0233-1 | Google Scholar 30442946 PMC6748154

[B18] Beijer D. Agnew T. Rack J. G. M. Prokhorova E. Deconinck T. Ceulemans B. (2021). Biallelic ADPRHL2 Mutations in Complex Neuropathy Affect ADP Ribosylation and DNA Damage Response. Life Sci. Alliance 4 (11), e202101057. 10.26508/lsa.202101057 PubMed Abstract | 10.26508/lsa.202101057 | Google Scholar 34479984 PMC8424258

[B19] Belousova E. A. Ishchenko А. A. Lavrik O. I. (2018). Dna Is a New Target of Parp3. Sci. Rep. 8 (1), 4176. 10.1038/s41598-018-22673-3 PubMed Abstract | 10.1038/s41598-018-22673-3 | Google Scholar 29520010 PMC5843604

[B20] Berger W. Steiner E. Grusch M. Elbling L. Micksche M. (2009). Vaults and the Major Vault Protein: Novel Roles in Signal Pathway Regulation and Immunity. Cell. Mol. Life Sci. 66 (1), 43–61. 10.1007/s00018-008-8364-z PubMed Abstract | 10.1007/s00018-008-8364-z | Google Scholar 18759128 PMC11131553

[B21] Bergeron-Sandoval L.-P. Safaee N. Michnick S. W. (2016). Mechanisms and Consequences of Macromolecular Phase Separation. Cell 165 (5), 1067–1079. 10.1016/j.cell.2016.05.026 PubMed Abstract | 10.1016/j.cell.2016.05.026 | Google Scholar 27203111

[B22] Bick M. J. Carroll J.-W. N. Gao G. Goff S. P. Rice C. M. MacDonald M. R. (2003). Expression of the Zinc-finger Antiviral Protein Inhibits Alphavirus Replication. J. Virol. 77 (21), 11555–11562. 10.1128/jvi.77.21.11555-11562.2003 PubMed Abstract | 10.1128/jvi.77.21.11555-11562.2003 | Google Scholar 14557641 PMC229374

[B23] Bilokapic S. Suskiewicz M. J. Ahel I. Halic M. (2020). Bridging of DNA Breaks Activates PARP2-HPF1 to Modify Chromatin. Nature 585 (7826), 609–613. 10.1038/s41586-020-2725-7 PubMed Abstract | 10.1038/s41586-020-2725-7 | Google Scholar 32939087 PMC7529888

[B24] Bonfiglio J. J. Fontana P. Zhang Q. Colby T. Gibbs-Seymour I. Atanassov I. (2017). Serine ADP-Ribosylation Depends on HPF1. Mol. Cel 65 (5), 932–940. 10.1016/j.molcel.2017.01.003 PubMed Abstract | 10.1016/j.molcel.2017.01.003 | Google Scholar PMC5344681 28190768

[B25] Bonfiglio J. J. Leidecker O. Dauben H. Longarini E. J. Colby T. San Segundo-Acosta P. (2020). An HPF1/PARP1-Based Chemical Biology Strategy for Exploring ADP-Ribosylation. Cell 183 (4), 1086–1102. 10.1016/j.cell.2020.09.055 PubMed Abstract | 10.1016/j.cell.2020.09.055 | Google Scholar 33186521

[B26] Boulikas T. (1990). Poly(ADP-ribosylated) Histones in Chromatin Replication. J. Biol. Chem. 265 (24), 14638–14647. 10.1016/s0021-9258(18)77350-x PubMed Abstract | 10.1016/s0021-9258(18)77350-x | Google Scholar 2387872

[B27] Brustel J. Muramoto T. Fumimoto K. Ellins J. Pears C. J. Lakin N. D. (2022). Linking DNA Repair and Cell Cycle Progression through Serine ADP-Ribosylation of Histones. Nat. Commun. 13 (1), 185. 10.1038/s41467-021-27867-4 PubMed Abstract | 10.1038/s41467-021-27867-4 | Google Scholar 35027540 PMC8758696

[B28] Buch-Larsen S. C. Hendriks I. A. Lodge J. M. Rykær M. Furtwängler B. Shishkova E. (2020). Mapping Physiological ADP-Ribosylation Using Activated Ion Electron Transfer Dissociation. Cel Rep. 32 (12), 108176. 10.1016/j.celrep.2020.108176 PubMed Abstract | 10.1016/j.celrep.2020.108176 | Google Scholar PMC7508052 32966781

[B29] Bütepage M. Krieg S. Eckei L. Li J. Rossetti G. Verheugd P. (2018). Assessment of Intracellular Auto-Modification Levels of ARTD10 Using Mono-ADP-ribose-specific Macrodomains 2 and 3 of Murine Artd8. Methods Mol. Biol. 1813, 41–63. 10.1007/978-1-4939-8588-3_4 PubMed Abstract | 10.1007/978-1-4939-8588-3_4 | Google Scholar 30097860

[B30] Cardamone M. D. Gao Y. Kwan J. Hayashi V. Sheeran M. Xu J. (2020). ADP-ribosylation of Mitochondrial Proteins Is Mediated by Neuralized-like Protein 4 (NEURL4). bioRxiv 221, e202101021. 10.1083/jcb.202101021 10.1083/jcb.202101021 | Google Scholar PMC8932523 35157000

[B31] Carter-O'Connell I. Jin H. Morgan R. K. David L. L. Cohen M. S. (2014). Engineering the Substrate Specificity of ADP-Ribosyltransferases for Identifying Direct Protein Targets. J. Am. Chem. Soc. 136 (14), 5201–5204. 10.1021/ja412897a PubMed Abstract | 10.1021/ja412897a | Google Scholar 24641686 PMC4445677

[B32] Carter-O'Connell I. Jin H. Morgan R. K. Zaja R. David L. L. Ahel I. (2016). Identifying Family-member-specific Targets of Mono-ARTDs by Using a Chemical Genetics Approach. Cell Rep 14 (3), 621–631. 10.1016/j.celrep.2015.12.045 PubMed Abstract | 10.1016/j.celrep.2015.12.045 | Google Scholar 26774478 PMC5423403

[B33] Carter-O'Connell I. Vermehren-Schmaedick A. Jin H. Morgan R. K. David L. L. Cohen M. S. (2018). Combining Chemical Genetics with Proximity-dependent Labeling Reveals Cellular Targets of Poly(ADP-Ribose) Polymerase 14 (PARP14). ACS Chem. Biol. 13 (10), 2841–2848. 10.1021/acschembio.8b00567 PubMed Abstract | 10.1021/acschembio.8b00567 | Google Scholar 30247868

[B34] Catara G. Grimaldi G. Schembri L. Spano D. Turacchio G. Lo Monte M. (2017). PARP1-produced Poly-ADP-Ribose Causes the PARP12 Translocation to Stress Granules and Impairment of Golgi Complex Functions. Sci. Rep. 7 (1), 14035. 10.1038/s41598-017-14156-8 PubMed Abstract | 10.1038/s41598-017-14156-8 | Google Scholar 29070863 PMC5656619

[B35] Challa S. Khulpateea B. R. Nandu T. Camacho C. V. Ryu K. W. Chen H. (2021). Ribosome ADP-Ribosylation Inhibits Translation and Maintains Proteostasis in Cancers. Cell 184 (17), 4531–4546. 10.1016/j.cell.2021.07.005 PubMed Abstract | 10.1016/j.cell.2021.07.005 | Google Scholar 34314702 PMC8380725

[B36] Chatrin C. Gabrielsen M. Buetow L. Nakasone M. A. Ahmed S. F. Sumpton D. (2020). Structural Insights into ADP-Ribosylation of Ubiquitin by Deltex Family E3 Ubiquitin Ligases. Sci. Adv. 6 (38), eabc0418. 10.1126/sciadv.abc0418 PubMed Abstract | 10.1126/sciadv.abc0418 | Google Scholar 32948590 PMC7500938

[B37] Chen D. Vollmar M. Rossi M. N. Phillips C. Kraehenbuehl R. Slade D. (2011). Identification of Macrodomain Proteins as Novel O-Acetyl-ADP-Ribose Deacetylases. J. Biol. Chem. 286 (15), 13261–13271. 10.1074/jbc.m110.206771 PubMed Abstract | 10.1074/jbc.m110.206771 | Google Scholar 21257746 PMC3075673

[B38] Chen X. Cubillos-Ruiz J. R. (2021). Endoplasmic Reticulum Stress Signals in the Tumour and its Microenvironment. Nat. Rev. Cancer 21 (2), 71–88. 10.1038/s41568-020-00312-2 PubMed Abstract | 10.1038/s41568-020-00312-2 | Google Scholar 33214692 PMC7927882

[B39] Cheon H. Borden E. C. Stark G. R. (2014). Interferons and Their Stimulated Genes in the Tumor Microenvironment. Semin. Oncol. 41 (2), 156–173. 10.1053/j.seminoncol.2014.02.002 PubMed Abstract | 10.1053/j.seminoncol.2014.02.002 | Google Scholar 24787290 PMC4118773

[B40] Cho K. F. Branon T. C. Udeshi N. D. Myers S. A. Carr S. A. Ting A. Y. (2020). Proximity Labeling in Mammalian Cells with TurboID and Split-TurboID. Nat. Protoc. 15 (12), 3971–3999. 10.1038/s41596-020-0399-0 PubMed Abstract | 10.1038/s41596-020-0399-0 | Google Scholar 33139955

[B41] Choudhary C. Kumar C. Gnad F. Nielsen M. L. Rehman M. Walther T. C. (2009). Lysine Acetylation Targets Protein Complexes and Co-regulates Major Cellular Functions. Science 325 (5942), 834–840. 10.1126/science.1175371 PubMed Abstract | 10.1126/science.1175371 | Google Scholar 19608861

[B42] Cohen M. S. (2020). Interplay between Compartmentalized NAD+ Synthesis and Consumption: a Focus on the PARP Family. Genes Dev. 34 (5-6), 254–262. 10.1101/gad.335109.119 PubMed Abstract | 10.1101/gad.335109.119 | Google Scholar 32029457 PMC7050480

[B43] Cohen M. S. Chang P. (2018). Insights into the Biogenesis, Function, and Regulation of ADP-Ribosylation. Nat. Chem. Biol. 14 (3), 236–243. 10.1038/nchembio.2568 PubMed Abstract | 10.1038/nchembio.2568 | Google Scholar 29443986 PMC5922452

[B44] Crawford K. Bonfiglio J. J. Mikoč A. Matic I. Ahel I. (2018). Specificity of Reversible ADP-Ribosylation and Regulation of Cellular Processes. Crit. Rev. Biochem. Mol. Biol. 53 (1), 64–82. 10.1080/10409238.2017.1394265 PubMed Abstract | 10.1080/10409238.2017.1394265 | Google Scholar 29098880

[B45] Cybulsky A. V. (2017). Endoplasmic Reticulum Stress, the Unfolded Protein Response and Autophagy in Kidney Diseases. Nat. Rev. Nephrol. 13 (11), 681–696. 10.1038/nrneph.2017.129 PubMed Abstract | 10.1038/nrneph.2017.129 | Google Scholar 28970584

[B46] Dani N. Stilla A. Marchegiani A. Tamburro A. Till S. Ladurner A. G. (2009). Combining Affinity Purification by ADP-Ribose-Binding Macro Domains with Mass Spectrometry to Define the Mammalian ADP-Ribosyl Proteome. Proc. Natl. Acad. Sci. U.S.A. 106 (11), 4243–4248. 10.1073/pnas.0900066106 PubMed Abstract | 10.1073/pnas.0900066106 | Google Scholar 19246377 PMC2657436

[B47] Daniels C. M. Ong S.-E. Leung A. K. L. (2014). Phosphoproteomic Approach to Characterize Protein Mono- and poly(ADP-Ribosyl)ation Sites from Cells. J. Proteome Res. 13 (8), 3510–3522. 10.1021/pr401032q PubMed Abstract | 10.1021/pr401032q | Google Scholar 24920161 PMC4123941

[B48] Daniels C. M. Thirawatananond P. Ong S.-E. Gabelli S. B. Leung A. K. L. (2015). Nudix Hydrolases Degrade Protein-Conjugated ADP-Ribose. Sci. Rep. 5, 18271. 10.1038/srep18271 PubMed Abstract | 10.1038/srep18271 | Google Scholar 26669448 PMC4680915

[B49] Dhoonmoon A. Schleicher E. M. Clements K. E. Nicolae C. M. Moldovan G. L. (2020). Genome-wide CRISPR Synthetic Lethality Screen Identifies a Role for the ADP-Ribosyltransferase PARP14 in DNA Replication Dynamics Controlled by ATR. Nucleic Acids Res. 48 (13), 7252–7264. 10.1093/nar/gkaa508 PubMed Abstract | 10.1093/nar/gkaa508 | Google Scholar 32542389 PMC7367200

[B50] Di Lisa F. Menabò R. Canton M. Barile M. Bernardi P. (2001). Opening of the Mitochondrial Permeability Transition Pore Causes Depletion of Mitochondrial and Cytosolic NAD+and Is a Causative Event in the Death of Myocytes in Postischemic Reperfusion of the Heart. J. Biol. Chem. 276 (4), 2571–2575. 10.1074/jbc.m006825200 PubMed Abstract | 10.1074/jbc.m006825200 | Google Scholar 11073947

[B51] Di Paola S. Micaroni M. Di Tullio G. Buccione R. Di Girolamo M. (2012). PARP16/ARTD15 Is a Novel Endoplasmic-Reticulum-Associated Mono-ADP-Ribosyltransferase that Interacts with, and Modifies Karyopherin-SS1. PLoS One 7 (6), e37352. 10.1371/journal.pone.0037352 PubMed Abstract | 10.1371/journal.pone.0037352 | Google Scholar 22701565 PMC3372510

[B52] Dölle C. Ziegler M. (2017). ADP-ribosylation of DNA Moving into Focus. Febs j 284 (23), 3999–4001. 10.1111/febs.14326 PubMed Abstract | 10.1111/febs.14326 | Google Scholar 29205912

[B53] El-Khamisy S. F. Masutani M. Suzuki H. Caldecott K. W. (2003). A Requirement for PARP-1 for the Assembly or Stability of XRCC1 Nuclear Foci at Sites of Oxidative DNA Damage. Nucleic Acids Res. 31 (19), 5526–5533. 10.1093/nar/gkg761 PubMed Abstract | 10.1093/nar/gkg761 | Google Scholar 14500814 PMC206461

[B54] Fabrizio G. Di Paola S. Stilla A. Giannotta M. Ruggiero C. Menzel S. (2015). ARTC1-mediated ADP-Ribosylation of GRP78/BiP: a New Player in Endoplasmic-Reticulum Stress Responses. Cel. Mol. Life Sci. 72 (6), 1209–1225. 10.1007/s00018-014-1745-6 PubMed Abstract | 10.1007/s00018-014-1745-6 | Google Scholar PMC11113179 25292337

[B55] Fenton A. L. Shirodkar P. Macrae C. J. Meng L. Koch C. A. (2013). The PARP3- and ATM-dependent Phosphorylation of APLF Facilitates DNA Double-Strand Break Repair. Nucleic Acids Res. 41 (7), 4080–4092. 10.1093/nar/gkt134 PubMed Abstract | 10.1093/nar/gkt134 | Google Scholar 23449221 PMC3627606

[B56] Fontana P. Bonfiglio J. J. Palazzo L. Bartlett E. Matic I. Ahel I. (2017). Serine ADP-Ribosylation Reversal by the Hydrolase ARH3. Elife 6, e28533. 10.7554/eLife.28533 PubMed Abstract | 10.7554/eLife.28533 | Google Scholar 28650317 PMC5552275

[B57] Forst A. H. Karlberg T. Herzog N. Thorsell A.-G. Gross A. Feijs K. L. H. (2013). Recognition of Mono-ADP-Ribosylated ARTD10 Substrates by ARTD8 Macrodomains. Structure 21 (3), 462–475. 10.1016/j.str.2012.12.019 PubMed Abstract | 10.1016/j.str.2012.12.019 | Google Scholar 23473667

[B58] Frese C. K. Zhou H. Taus T. Altelaar A. F. M. Mechtler K. Heck A. J. R. (2013). Unambiguous Phosphosite Localization Using Electron-Transfer/higher-Energy Collision Dissociation (EThcD). J. Proteome Res. 12 (3), 1520–1525. 10.1021/pr301130k PubMed Abstract | 10.1021/pr301130k | Google Scholar 23347405 PMC3588588

[B59] Gagné J.-P. Pic É. Isabelle M. Krietsch J. Éthier C. Paquet É. (2012). Quantitative Proteomics Profiling of the poly(ADP-Ribose)-Related Response to Genotoxic Stress. Nucleic Acids Res. 40 (16), 7788–7805. 10.1093/nar/gks486 PubMed Abstract | 10.1093/nar/gks486 | Google Scholar 22669911 PMC3439892

[B60] García-Saura A. G. Herzog L. K. Dantuma N. P. Schüler H. (2021). MacroGreen, a Simple Tool for Detection of ADP-Ribosylated Proteins. Commun. Biol. 4 (1), 919. 10.1038/s42003-021-02439-w PubMed Abstract | 10.1038/s42003-021-02439-w | Google Scholar 34321589 PMC8319303

[B61] Gibson B. A. Conrad L. B. Huang D. Kraus W. L. (2017). Generation and Characterization of Recombinant Antibody-like ADP-Ribose Binding Proteins. Biochemistry 56 (48), 6305–6316. 10.1021/acs.biochem.7b00670 PubMed Abstract | 10.1021/acs.biochem.7b00670 | Google Scholar 29053245 PMC6465537

[B62] Gibson B. A. Kraus W. L. (2017). Identification of Protein Substrates of Specific PARP Enzymes Using Analog-Sensitive PARP Mutants and a "Clickable" NAD+ Analog. Methods Mol. Biol. 1608, 111–135. 10.1007/978-1-4939-6993-7_9 PubMed Abstract | 10.1007/978-1-4939-6993-7_9 | Google Scholar 28695507 PMC6465536

[B63] Gibson B. A. Kraus W. L. (2012). New Insights into the Molecular and Cellular Functions of poly(ADP-Ribose) and PARPs. Nat. Rev. Mol. Cel Biol 13 (7), 411–424. 10.1038/nrm3376 PubMed Abstract | 10.1038/nrm3376 | Google Scholar 22713970

[B64] Gibson B. A. Zhang Y. Jiang H. Hussey K. M. Shrimp J. H. Lin H. (2016). Chemical Genetic Discovery of PARP Targets Reveals a Role for PARP-1 in Transcription Elongation. Science 353 (6294), 45–50. 10.1126/science.aaf7865 PubMed Abstract | 10.1126/science.aaf7865 | Google Scholar 27256882 PMC5540732

[B65] Gomez A. Bindesbøll C. Satheesh S. V. Grimaldi G. Hutin D. MacPherson L. (2018). Characterization of TCDD-Inducible Poly-ADP-Ribose Polymerase (TIPARP/ARTD14) Catalytic Activity. Biochem. J. 475 (23), 3827–3846. 10.1042/bcj20180347 PubMed Abstract | 10.1042/bcj20180347 | Google Scholar 30373764 PMC6292455

[B66] Gozgit J. M. Vasbinder M. M. Abo R. P. Kunii K. Kuplast-Barr K. G. Gui B. (2021). PARP7 Negatively Regulates the Type I Interferon Response in Cancer Cells and its Inhibition Triggers Antitumor Immunity. Cancer Cell 39 (9), 1214–1226. 10.1016/j.ccell.2021.06.018 PubMed Abstract | 10.1016/j.ccell.2021.06.018 | Google Scholar 34375612

[B67] Groslambert J. Prokhorova E. Ahel I. (2021). ADP-ribosylation of DNA and RNA. DNA Repair 105, 103144. 10.1016/j.dnarep.2021.103144 PubMed Abstract | 10.1016/j.dnarep.2021.103144 | Google Scholar 34116477 PMC8385414

[B68] Guo T. Zuo Y. Qian L. Liu J. Yuan Y. Xu K. (2019). ADP-ribosyltransferase PARP11 Modulates the Interferon Antiviral Response by Mono-ADP-Ribosylating the Ubiquitin E3 Ligase β-TrCP. Nat. Microbiol. 4 (11), 1872–1884. 10.1038/s41564-019-0428-3 PubMed Abstract | 10.1038/s41564-019-0428-3 | Google Scholar 30988430

[B69] Guo X. Carroll J.-W. N. Macdonald M. R. Goff S. P. Gao G. (2004). The Zinc finger Antiviral Protein Directly Binds to Specific Viral mRNAs through the CCCH Zinc finger Motifs. J. Virol. 78 (23), 12781–12787. 10.1128/jvi.78.23.12781-12787.2004 PubMed Abstract | 10.1128/jvi.78.23.12781-12787.2004 | Google Scholar 15542630 PMC525010

[B70] Guo X. Ma J. Sun J. Gao G. (2007). The Zinc-finger Antiviral Protein Recruits the RNA Processing Exosome to Degrade the Target mRNA. Proc. Natl. Acad. Sci. U.S.A. 104 (1), 151–156. 10.1073/pnas.0607063104 PubMed Abstract | 10.1073/pnas.0607063104 | Google Scholar 17185417 PMC1765426

[B71] Gupte R. Liu Z. Kraus W. L. (2017). PARPs and ADP-Ribosylation: Recent Advances Linking Molecular Functions to Biological Outcomes. Genes Dev. 31 (2), 101–126. 10.1101/gad.291518.116 PubMed Abstract | 10.1101/gad.291518.116 | Google Scholar 28202539 PMC5322727

[B72] Guthals A. Bandeira N. (2012). Peptide Identification by Tandem Mass Spectrometry with Alternate Fragmentation Modes. Mol. Cell Proteomics 11 (9), 550–557. 10.1074/mcp.r112.018556 PubMed Abstract | 10.1074/mcp.r112.018556 | Google Scholar 22595789 PMC3434779

[B73] Härtlova A. Erttmann S. F. Raffi F. A. Schmalz A. M. Resch U. Anugula S. (2015). DNA Damage Primes the Type I Interferon System via the Cytosolic DNA Sensor STING to Promote Anti-microbial Innate Immunity. Immunity 42 (2), 332–343. 10.1016/j.immuni.2015.01.012 PubMed Abstract | 10.1016/j.immuni.2015.01.012 | Google Scholar 25692705

[B74] He F. Tsuda K. Takahashi M. Kuwasako K. Terada T. Shirouzu M. (2012). Structural Insight into the Interaction of ADP-Ribose with the PARP WWE Domains. FEBS Lett. 586 (21), 3858–3864. 10.1016/j.febslet.2012.09.009 PubMed Abstract | 10.1016/j.febslet.2012.09.009 | Google Scholar 23010590

[B75] Helleday T. (2011). The Underlying Mechanism for the PARP and BRCA Synthetic Lethality: Clearing up the Misunderstandings. Mol. Oncol. 5 (4), 387–393. 10.1016/j.molonc.2011.07.001 PubMed Abstract | 10.1016/j.molonc.2011.07.001 | Google Scholar 21821475 PMC5528309

[B76] Higashi H. Maejima T. Lee L. H. Yamazaki Y. Hottiger M. O. Singh S. A. (2019). A Study into the ADP-Ribosylome of IFN-γ-Stimulated THP-1 Human Macrophage-like Cells Identifies ARTD8/PARP14 and ARTD9/PARP9 ADP-Ribosylation. J. Proteome Res. 18 (4), 1607–1622. 10.1021/acs.jproteome.8b00895 PubMed Abstract | 10.1021/acs.jproteome.8b00895 | Google Scholar 30848916 PMC6456868

[B77] Hopp A.-K. Teloni F. Bisceglie L. Gondrand C. Raith F. Nowak K. (2021). Mitochondrial NAD+ Controls Nuclear ARTD1-Induced ADP-Ribosylation. Mol. Cel 81 (2), 340–354. 10.1016/j.molcel.2020.12.034 10.1016/j.molcel.2020.12.034 | Google Scholar PMC7837215 33450210

[B78] Hopp A. K. Hottiger M. O. (2021). Uncovering the Invisible: Mono-ADP-Ribosylation Moved into the Spotlight. Cells 10 (3), 680. 10.3390/cells10030680 PubMed Abstract | 10.3390/cells10030680 | Google Scholar 33808662 PMC8003356

[B79] Hottiger M. O. Hassa P. O. Lüscher B. Schüler H. Koch-Nolte F. (2010). Toward a Unified Nomenclature for Mammalian ADP-Ribosyltransferases. Trends Biochem. Sci. 35 (4), 208–219. 10.1016/j.tibs.2009.12.003 PubMed Abstract | 10.1016/j.tibs.2009.12.003 | Google Scholar 20106667

[B80] Iansante V. Choy P. M. Fung S. W. Liu Y. Chai J.-G. Dyson J. (2015). PARP14 Promotes the Warburg Effect in Hepatocellular Carcinoma by Inhibiting JNK1-dependent PKM2 Phosphorylation and Activation. Nat. Commun. 6, 7882. 10.1038/ncomms8882 PubMed Abstract | 10.1038/ncomms8882 | Google Scholar 26258887 PMC4918319

[B81] Ivashkiv L. B. Donlin L. T. (2014). Regulation of Type I Interferon Responses. Nat. Rev. Immunol. 14 (1), 36–49. 10.1038/nri3581 PubMed Abstract | 10.1038/nri3581 | Google Scholar 24362405 PMC4084561

[B82] Iwata H. Goettsch C. Sharma A. Ricchiuto P. Goh W. W. B. Halu A. (2016). PARP9 and PARP14 Cross-Regulate Macrophage Activation via STAT1 ADP-Ribosylation. Nat. Commun. 7, 12849. 10.1038/ncomms12849 PubMed Abstract | 10.1038/ncomms12849 | Google Scholar 27796300 PMC5095532

[B83] Jankevicius G. Hassler M. Golia B. Rybin V. Zacharias M. Timinszky G. (2013). A Family of Macrodomain Proteins Reverses Cellular Mono-ADP-Ribosylation. Nat. Struct. Mol. Biol. 20 (4), 508–514. 10.1038/nsmb.2523 PubMed Abstract | 10.1038/nsmb.2523 | Google Scholar 23474712 PMC7097781

[B84] Jiao S. Xia W. Yamaguchi H. Wei Y. Chen M.-K. Hsu J.-M. (2017). PARP Inhibitor Upregulates PD-L1 Expression and Enhances Cancer-Associated Immunosuppression. Clin. Cancer Res. 23 (14), 3711–3720. 10.1158/1078-0432.ccr-16-3215 PubMed Abstract | 10.1158/1078-0432.ccr-16-3215 | Google Scholar 28167507 PMC5511572

[B85] Jungmichel S. Rosenthal F. Altmeyer M. Lukas J. Hottiger M. O. Nielsen M. L. (2013). Proteome-wide Identification of poly(ADP-Ribosyl)ation Targets in Different Genotoxic Stress Responses. Mol. Cel 52 (2), 272–285. 10.1016/j.molcel.2013.08.026 PubMed Abstract | 10.1016/j.molcel.2013.08.026 | Google Scholar 24055347

[B86] Juszczynski P. Kutok J. L. Li C. Mitra J. Aguiar R. C. T. Shipp M. A. (2006). BAL1 and BBAP Are Regulated by a Gamma Interferon-Responsive Bidirectional Promoter and Are Overexpressed in Diffuse Large B-Cell Lymphomas with a Prominent Inflammatory Infiltrate. Mol. Cel Biol 26 (14), 5348–5359. 10.1128/mcb.02351-05 PubMed Abstract | 10.1128/mcb.02351-05 | Google Scholar PMC1592708 16809771

[B87] Jwa M. Chang P. (2012). PARP16 Is a Tail-Anchored Endoplasmic Reticulum Protein Required for the PERK- and IRE1α-Mediated Unfolded Protein Response. Nat. Cel Biol 14 (11), 1223–1230. 10.1038/ncb2593 10.1038/ncb2593 | Google Scholar PMC3494284 23103912

[B88] Kang H. C. Lee Y.-I. Shin J.-H. Andrabi S. A. Chi Z. Gagné J.-P. (2011). Iduna Is a poly(ADP-Ribose) (PAR)-dependent E3 Ubiquitin Ligase that Regulates DNA Damage. Proc. Natl. Acad. Sci. U.S.A. 108 (34), 14103–14108. 10.1073/pnas.1108799108 PubMed Abstract | 10.1073/pnas.1108799108 | Google Scholar 21825151 PMC3161609

[B89] Karch K. R. Langelier M.-F. Pascal J. M. Garcia B. A. (2017). The Nucleosomal Surface Is the Main Target of Histone ADP-Ribosylation in Response to DNA Damage. Mol. Biosyst. 13 (12), 2660–2671. 10.1039/c7mb00498b PubMed Abstract | 10.1039/c7mb00498b | Google Scholar 29058739 PMC5702540

[B90] Karlberg T. Klepsch M. Thorsell A.-G. Andersson C. D. Linusson A. Schüler H. (2015). Structural Basis for Lack of ADP-Ribosyltransferase Activity in poly(ADP-Ribose) Polymerase-13/zinc finger Antiviral Protein. J. Biol. Chem. 290 (12), 7336–7344. 10.1074/jbc.m114.630160 PubMed Abstract | 10.1074/jbc.m114.630160 | Google Scholar 25635049 PMC4367243

[B91] Karlberg T. Thorsell A.-G. Kallas Å. Schüler H. (2012). Crystal Structure of Human ADP-Ribose Transferase ARTD15/PARP16 Reveals a Novel Putative Regulatory Domain. J. Biol. Chem. 287 (29), 24077–24081. 10.1074/jbc.m112.379289 PubMed Abstract | 10.1074/jbc.m112.379289 | Google Scholar 22661712 PMC3397834

[B92] Karras G. I. Kustatscher G. Buhecha H. R. Allen M. D. Pugieux C. Sait F. (2005). The Macro Domain Is an ADP-Ribose Binding Module. Embo j 24 (11), 1911–1920. 10.1038/sj.emboj.7600664 PubMed Abstract | 10.1038/sj.emboj.7600664 | Google Scholar 15902274 PMC1142602

[B93] Kawamitsu H. Hoshino H. Okada H. Miwa M. Momoi H. Sugimura T. (1984). Monoclonal Antibodies to Poly(adenosine Diphosphate Ribose) Recognize Different Structures. Biochemistry 23 (16), 3771–3777. 10.1021/bi00311a032 PubMed Abstract | 10.1021/bi00311a032 | Google Scholar 6206890

[B94] Kim D.-S. Camacho C. V. Nagari A. Malladi V. S. Challa S. Kraus W. L. (2019). Activation of PARP-1 by snoRNAs Controls Ribosome Biogenesis and Cell Growth via the RNA Helicase DDX21. Mol. Cel 75 (6), 1270–1285. 10.1016/j.molcel.2019.06.020 PubMed Abstract | 10.1016/j.molcel.2019.06.020 | Google Scholar PMC6754283 31351877

[B95] Kim I.-K. Stegeman R. A. Brosey C. A. Ellenberger T. (2015). A Quantitative Assay Reveals Ligand Specificity of the DNA Scaffold Repair Protein XRCC1 and Efficient Disassembly of Complexes of XRCC1 and the poly(ADP-Ribose) Polymerase 1 by poly(ADP-Ribose) Glycohydrolase. J. Biol. Chem. 290 (6), 3775–3783. 10.1074/jbc.m114.624718 PubMed Abstract | 10.1074/jbc.m114.624718 | Google Scholar 25477519 PMC4319041

[B96] Kim M. Y. Mauro S. Gévry N. Lis J. T. Kraus W. L. (2004). NAD+-dependent Modulation of Chromatin Structure and Transcription by Nucleosome Binding Properties of PARP-1. Cell 119 (6), 803–814. 10.1016/j.cell.2004.11.002 PubMed Abstract | 10.1016/j.cell.2004.11.002 | Google Scholar 15607977

[B97] Kirby I. T. Kojic A. Arnold M. R. Thorsell A.-G. Karlberg T. Vermehren-Schmaedick A. (2018). A Potent and Selective PARP11 Inhibitor Suggests Coupling between Cellular Localization and Catalytic Activity. Cel Chem. Biol. 25 (12), 1547–1553. 10.1016/j.chembiol.2018.09.011 PubMed Abstract | 10.1016/j.chembiol.2018.09.011 | Google Scholar 30344052

[B98] Kirby I. T. Person A. Cohen M. (2021). Rational Design of Selective Inhibitors of PARP4. RSC Med. Chem. 12 (11), 1950–1957. 10.1039/d1md00195g PubMed Abstract | 10.1039/d1md00195g | Google Scholar 34825190 PMC8597433

[B99] Kliza K. W. Liu Q. Roosenboom L. W. M. Jansen P. Filippov D. V. Vermeulen M. (2021). Reading ADP-Ribosylation Signaling Using Chemical Biology and Interaction Proteomics. Mol. Cel. 81 (21), 4552–4567.e8. 10.1016/j.molcel.2021.08.037 10.1016/j.molcel.2021.08.037 | Google Scholar 34551281

[B100] Koch-Nolte F. Adriouch S. Bannas P. Krebs C. Scheuplein F. Seman M. (2006). ADP-ribosylation of Membrane Proteins: Unveiling the Secrets of a Crucial Regulatory Mechanism in Mammalian Cells. Ann. Med. 38 (3), 188–199. 10.1080/07853890600655499 PubMed Abstract | 10.1080/07853890600655499 | Google Scholar 16720433

[B101] Kors S. Geijtenbeek K. Reits E. Schipper-Krom S. (2019). Regulation of Proteasome Activity by (Post-)transcriptional Mechanisms. Front. Mol. Biosci. 6, 48. 10.3389/fmolb.2019.00048 PubMed Abstract | 10.3389/fmolb.2019.00048 | Google Scholar 31380390 PMC6646590

[B102] Kozaki T. Komano J. Kanbayashi D. Takahama M. Misawa T. Satoh T. (2017). Mitochondrial Damage Elicits a TCDD-Inducible poly(ADP-Ribose) Polymerase-Mediated Antiviral Response. Proc. Natl. Acad. Sci. U.S.A. 114 (10), 2681–2686. 10.1073/pnas.1621508114 PubMed Abstract | 10.1073/pnas.1621508114 | Google Scholar 28213497 PMC5347618

[B103] Krishnakumar R. Kraus W. L. (2010). PARP-1 Regulates Chromatin Structure and Transcription through a KDM5B-dependent Pathway. Mol. Cel 39 (5), 736–749. 10.1016/j.molcel.2010.08.014 PubMed Abstract | 10.1016/j.molcel.2010.08.014 | Google Scholar PMC2939044 20832725

[B104] Langelier M.-F. Billur R. Sverzhinsky A. Black B. E. Pascal J. M. (2021). HPF1 Dynamically Controls the PARP1/2 Balance between Initiating and Elongating ADP-Ribose Modifications. Nat. Commun. 12 (1), 6675. 10.1038/s41467-021-27043-8 PubMed Abstract | 10.1038/s41467-021-27043-8 | Google Scholar 34795260 PMC8602370

[B105] Langelier M.-F. Planck J. L. Roy S. Pascal J. M. (2012). Structural Basis for DNA Damage-dependent poly(ADP-Ribosyl)ation by Human PARP-1. Science 336 (6082), 728–732. 10.1126/science.1216338 PubMed Abstract | 10.1126/science.1216338 | Google Scholar 22582261 PMC3532513

[B106] Larsen S. C. Leutert M. Bilan V. Martello R. Jungmichel S. Young C. (2017). Proteome-Wide Identification of *In Vivo* ADP-Ribose Acceptor Sites by Liquid Chromatography-Tandem Mass Spectrometry. Methods Mol. Biol. 1608, 149–162. 10.1007/978-1-4939-6993-7_11 PubMed Abstract | 10.1007/978-1-4939-6993-7_11 | Google Scholar 28695509

[B107] Lau C. Niere M. Ziegler M. (2009). The NMN/NaMN Adenylyltransferase (NMNAT) Protein Family. Front. Biosci. 14, 410–431. 10.2741/3252 PubMed Abstract | 10.2741/3252 | Google Scholar 19273075

[B108] Leung A. K. L. Calabrese J. M. Sharp P. A. (2006). Quantitative Analysis of Argonaute Protein Reveals microRNA-dependent Localization to Stress Granules. Proc. Natl. Acad. Sci. U.S.A. 103 (48), 18125–18130. 10.1073/pnas.0608845103 PubMed Abstract | 10.1073/pnas.0608845103 | Google Scholar 17116888 PMC1838717

[B109] Leung A. K. L. Vyas S. Rood J. E. Bhutkar A. Sharp P. A. Chang P. (2011). Poly(ADP-ribose) Regulates Stress Responses and microRNA Activity in the Cytoplasm. Mol. Cel 42 (4), 489–499. 10.1016/j.molcel.2011.04.015 PubMed Abstract | 10.1016/j.molcel.2011.04.015 | Google Scholar PMC3898460 21596313

[B110] Liu L. Su X. Quinn W. J. 3rd Hui S. Krukenberg K. Frederick D. W. (2018). Quantitative Analysis of NAD Synthesis-Breakdown Fluxes. Cel Metab. 27 (5), 1067–1080. 10.1016/j.cmet.2018.03.018 PubMed Abstract | 10.1016/j.cmet.2018.03.018 | Google Scholar PMC5932087 29685734

[B111] Lu A. Z. Abo R. Ren Y. Gui B. Mo J.-R. Blackwell D. (2019). Enabling Drug Discovery for the PARP Protein Family through the Detection of Mono-ADP-Ribosylation. Biochem. Pharmacol. 167, 97–106. 10.1016/j.bcp.2019.05.007 PubMed Abstract | 10.1016/j.bcp.2019.05.007 | Google Scholar 31075269

[B112] Lu X. Alam U. Willis C. Kennedy D. (2021). Role of Chikungunya nsP3 in Regulating G3BP1 Activity, Stress Granule Formation and Drug Efficacy. Arch. Med. Res. 52 (1), 48–57. 10.1016/j.arcmed.2020.10.002 PubMed Abstract | 10.1016/j.arcmed.2020.10.002 | Google Scholar 33131924

[B113] Luo X. Kraus W. L. (2012). On PAR with PARP: Cellular Stress Signaling through poly(ADP-Ribose) and PARP-1. Genes Dev. 26 (5), 417–432. 10.1101/gad.183509.111 PubMed Abstract | 10.1101/gad.183509.111 | Google Scholar 22391446 PMC3305980

[B114] Lüscher B. Ahel I. Altmeyer M. Ashworth A. Bai P. Chang P. (2021). ADP-ribosyltransferases, an Update on Function and Nomenclature. FEBS J. Epub ahead of print. 10.1111/febs.16142 10.1111/febs.16142 | Google Scholar PMC9027952 34323016

[B115] Mackenzie K. J. Carroll P. Martin C.-A. Murina O. Fluteau A. Simpson D. J. (2017). cGAS Surveillance of Micronuclei Links Genome Instability to Innate Immunity. Nature 548 (7668), 461–465. 10.1038/nature23449 PubMed Abstract | 10.1038/nature23449 | Google Scholar 28738408 PMC5870830

[B116] Maksimainen M. M. Murthy S. Sowa S. T. Galera-Prat A. Rolina E. Heiskanen J. P. (2021). Analogs of TIQ-A as Inhibitors of Human Mono-ADP-Ribosylating PARPs. Bioorg. Med. Chem. 52, 116511. 10.1016/j.bmc.2021.116511 PubMed Abstract | 10.1016/j.bmc.2021.116511 | Google Scholar 34801828

[B117] Marcus J. M. Hossain M. I. Gagné J.-P. Poirier G. G. McMahon L. L. Cowell R. M. (2021). PARP-1 Activation Leads to Cytosolic Accumulation of TDP-43 in Neurons. Neurochem. Int. 148, 105077. 10.1016/j.neuint.2021.105077 PubMed Abstract | 10.1016/j.neuint.2021.105077 | Google Scholar 34082062

[B118] Martello R. Leutert M. Jungmichel S. Bilan V. Larsen S. C. Young C. (2016). Proteome-wide Identification of the Endogenous ADP-Ribosylome of Mammalian Cells and Tissue. Nat. Commun. 7, 12917. 10.1038/ncomms12917 PubMed Abstract | 10.1038/ncomms12917 | Google Scholar 27686526 PMC5056437

[B119] Masutani M. Fujimori H. (2013). Poly(ADP-ribosyl)ation in Carcinogenesis. Mol. Aspects Med. 34 (6), 1202–1216. 10.1016/j.mam.2013.05.003 PubMed Abstract | 10.1016/j.mam.2013.05.003 | Google Scholar 23714734

[B120] McCormick C. Khaperskyy D. A. (2017). Translation Inhibition and Stress Granules in the Antiviral Immune Response. Nat. Rev. Immunol. 17 (10), 647–660. 10.1038/nri.2017.63 PubMed Abstract | 10.1038/nri.2017.63 | Google Scholar 28669985

[B121] Mehrotra P. Riley J. P. Patel R. Li F. Voss L. e. Goenka S. (2011). PARP-14 Functions as a Transcriptional Switch for Stat6-dependent Gene Activation. J. Biol. Chem. 286 (3), 1767–1776. 10.1074/jbc.m110.157768 PubMed Abstract | 10.1074/jbc.m110.157768 | Google Scholar 21081493 PMC3023471

[B122] Molina H. Horn D. M. Tang N. Mathivanan S. Pandey A. (2007). Global Proteomic Profiling of Phosphopeptides Using Electron Transfer Dissociation Tandem Mass Spectrometry. Proc. Natl. Acad. Sci. U.S.A. 104 (7), 2199–2204. 10.1073/pnas.0611217104 PubMed Abstract | 10.1073/pnas.0611217104 | Google Scholar 17287340 PMC1794346

[B123] Morgan R. K. Cohen M. S. (2015). A Clickable Aminooxy Probe for Monitoring Cellular ADP-Ribosylation. ACS Chem. Biol. 10 (8), 1778–1784. 10.1021/acschembio.5b00213 PubMed Abstract | 10.1021/acschembio.5b00213 | Google Scholar 25978521 PMC4546562

[B124] Morgan R. K. Cohen M. S. (2017). “Detecting Protein ADP-Ribosylation Using a Clickable Aminooxy Probe,” in Poly(ADP-Ribose) Polymerase: Methods and Protocols. Editor Tulin AV (New York, NY: Springer New York), 71–77. 10.1007/978-1-4939-6993-7_6 PubMed Abstract | 10.1007/978-1-4939-6993-7_6 | Google Scholar PMC5920556 28695504

[B125] Moss J. Stanley S. J. Nightingale M. S. Murtagh J. J. Jr. Monaco L. Mishima K. (1992). Molecular and Immunological Characterization of ADP-Ribosylarginine Hydrolases. J. Biol. Chem. 267 (15), 10481–10488. 10.1016/s0021-9258(19)50043-6 PubMed Abstract | 10.1016/s0021-9258(19)50043-6 | Google Scholar 1375222

[B126] Mossink M. H. van Zon A. Scheper R. J. Sonneveld P. Wiemer E. A. (2003). Vaults: a Ribonucleoprotein Particle Involved in Drug Resistance? Oncogene 22 (47), 7458–7467. 10.1038/sj.onc.1206947 PubMed Abstract | 10.1038/sj.onc.1206947 | Google Scholar 14576851

[B127] Moustakim M. Riedel K. Schuller M. Gehring A. P. Monteiro O. P. Martin S. P. (2018). Discovery of a Novel Allosteric Inhibitor Scaffold for Polyadenosine-Diphosphate-Ribose Polymerase 14 (PARP14) Macrodomain 2. Bioorg. Med. Chem. 26 (11), 2965–2972. 10.1016/j.bmc.2018.03.020 PubMed Abstract | 10.1016/j.bmc.2018.03.020 | Google Scholar 29567296 PMC6008491

[B128] Munnur D. Bartlett E. Mikolčević P. Kirby I. T. Rack J. G. M. Mikoč A. (2019). Reversible ADP-Ribosylation of RNA. Nucleic Acids Res. 47 (11), 5658–5669. 10.1093/nar/gkz305 PubMed Abstract | 10.1093/nar/gkz305 | Google Scholar 31216043 PMC6582358

[B129] Murthy S. Desantis J. Verheugd P. Maksimainen M. M. Venkannagari H. Massari S. (2018). 4-(Phenoxy) and 4-(benzyloxy)benzamides as Potent and Selective Inhibitors of Mono-ADP-Ribosyltransferase PARP10/ARTD10. Eur. J. Med. Chem. 156, 93–102. 10.1016/j.ejmech.2018.06.047 PubMed Abstract | 10.1016/j.ejmech.2018.06.047 | Google Scholar 30006177

[B130] Nagaraj N. Wisniewski J. R. Geiger T. Cox J. Kircher M. Kelso J. (2011). Deep Proteome and Transcriptome Mapping of a Human Cancer Cell Line. Mol. Syst. Biol. 7, 548. 10.1038/msb.2011.81 PubMed Abstract | 10.1038/msb.2011.81 | Google Scholar 22068331 PMC3261714

[B131] Nicolae C. M. Aho E. R. Choe K. N. Constantin D. Hu H.-J. Lee D. (2015). A Novel Role for the Mono-ADP-Ribosyltransferase PARP14/ARTD8 in Promoting Homologous Recombination and Protecting against Replication Stress. Nucleic Acids Res. 43 (6), 3143–3153. 10.1093/nar/gkv147 PubMed Abstract | 10.1093/nar/gkv147 | Google Scholar 25753673 PMC4381061

[B132] Nicolae C. M. Aho E. R. Vlahos A. H. S. Choe K. N. De S. Karras G. I. (2014). The ADP-Ribosyltransferase PARP10/ARTD10 Interacts with Proliferating Cell Nuclear Antigen (PCNA) and Is Required for DNA Damage Tolerance. J. Biol. Chem. 289 (19), 13627–13637. 10.1074/jbc.m114.556340 PubMed Abstract | 10.1074/jbc.m114.556340 | Google Scholar 24695737 PMC4036367

[B133] Niere M. Kernstock S. Koch-Nolte F. Ziegler M. (2008). Functional Localization of Two poly(ADP-Ribose)-Degrading Enzymes to the Mitochondrial Matrix. Mol. Cel Biol 28 (2), 814–824. 10.1128/mcb.01766-07 10.1128/mcb.01766-07 | Google Scholar PMC2223433 17991898

[B134] Niere M. Mashimo M. Agledal L. Dölle C. Kasamatsu A. Kato J. (2012). ADP-ribosylhydrolase 3 (ARH3), Not poly(ADP-Ribose) Glycohydrolase (PARG) Isoforms, Is Responsible for Degradation of Mitochondrial Matrix-Associated poly(ADP-Ribose). J. Biol. Chem. 287 (20), 16088–16102. 10.1074/jbc.m112.349183 PubMed Abstract | 10.1074/jbc.m112.349183 | Google Scholar 22433848 PMC3351285

[B135] Nowak K. Rosenthal F. Karlberg T. Bütepage M. Thorsell A.-G. Dreier B. (2020). Engineering Af1521 Improves ADP-Ribose Binding and Identification of ADP-Ribosylated Proteins. Nat. Commun. 11 (1), 5199. 10.1038/s41467-020-18981-w PubMed Abstract | 10.1038/s41467-020-18981-w | Google Scholar 33060572 PMC7566600

[B136] Olsen J. V. Vermeulen M. Santamaria A. Kumar C. Miller M. L. Jensen L. J. (2010). Quantitative Phosphoproteomics Reveals Widespread Full Phosphorylation Site Occupancy during Mitosis. Sci. Signal. 3 (104), ra3. 10.1126/scisignal.2000475 PubMed Abstract | 10.1126/scisignal.2000475 | Google Scholar 20068231

[B137] Palavalli Parsons L. H. Challa S. Gibson B. A. Nandu T. Stokes M. S. Huang D. (2021). Identification of PARP-7 Substrates Reveals a Role for MARylation in Microtubule Control in Ovarian Cancer Cells. Elife 10, e60481. 10.7554/eLife.60481 PubMed Abstract | 10.7554/eLife.60481 | Google Scholar 33475085 PMC7884071

[B138] Palazzo L. Daniels C. M. Nettleship J. E. Rahman N. McPherson R. L. Ong S. E. (2016). ENPP 1 Processes Protein ADP ‐ribosylation *In Vitro* . Febs j 283 (18), 3371–3388. 10.1111/febs.13811 PubMed Abstract | 10.1111/febs.13811 | Google Scholar 27406238 PMC5030157

[B139] Palazzo L. Thomas B. Jemth A.-S. Colby T. Leidecker O. Feijs K. L. H. (2015). Processing of Protein ADP-Ribosylation by Nudix Hydrolases. Biochem. J. 468 (2), 293–301. 10.1042/bj20141554 PubMed Abstract | 10.1042/bj20141554 | Google Scholar 25789582 PMC6057610

[B140] Paludan S. R. Reinert L. S. Hornung V. (2019). DNA-stimulated Cell Death: Implications for Host Defence, Inflammatory Diseases and Cancer. Nat. Rev. Immunol. 19 (3), 141–153. 10.1038/s41577-018-0117-0 PubMed Abstract | 10.1038/s41577-018-0117-0 | Google Scholar 30644449 PMC7311199

[B141] Pellegrino S. Altmeyer M. (2016). Interplay between Ubiquitin, SUMO, and Poly(ADP-Ribose) in the Cellular Response to Genotoxic Stress. Front. Genet. 7, 63. 10.3389/fgene.2016.00063 PubMed Abstract | 10.3389/fgene.2016.00063 | Google Scholar 27148359 PMC4835507

[B142] Perina D. Mikoč A. Ahel J. Ćetković H. Žaja R. Ahel I. (2014). Distribution of Protein poly(ADP-Ribosyl)ation Systems across All Domains of Life. DNA Repair 23, 4–16. 10.1016/j.dnarep.2014.05.003 PubMed Abstract | 10.1016/j.dnarep.2014.05.003 | Google Scholar 24865146 PMC4245714

[B143] Protter D. S. W. Parker R. (2016). Principles and Properties of Stress Granules. Trends Cel Biol. 26 (9), 668–679. 10.1016/j.tcb.2016.05.004 PubMed Abstract | 10.1016/j.tcb.2016.05.004 | Google Scholar PMC4993645 27289443

[B144] Qi G. Kudo Y. Tang B. Liu T. Jin S. Liu J. (2016). PARP6 Acts as a Tumor Suppressor via Downregulating Survivin Expression in Colorectal Cancer. Oncotarget 7 (14), 18812–18824. 10.18632/oncotarget.7712 PubMed Abstract | 10.18632/oncotarget.7712 | Google Scholar 26934315 PMC4951331

[B145] Qiu J. Sheedlo M. J. Yu K. Tan Y. Nakayasu E. S. Das C. (2016). Ubiquitination Independent of E1 and E2 Enzymes by Bacterial Effectors. Nature 533 (7601), 120–124. 10.1038/nature17657 PubMed Abstract | 10.1038/nature17657 | Google Scholar 27049943 PMC4905768

[B146] Rack J. G. M. Palazzo L. Ahel I. (2020). (ADP-ribosyl)hydrolases: Structure, Function, and Biology. Genes Dev. 34 (5-6), 263–284. 10.1101/gad.334631.119 PubMed Abstract | 10.1101/gad.334631.119 | Google Scholar 32029451 PMC7050489

[B147] Rack J. G. M. Liu Q. Zorzini V. Voorneveld J. Ariza A. Honarmand Ebrahimi K. (2021). Mechanistic Insights into the Three Steps of poly(ADP-Ribosylation) Reversal. Nat. Commun. 12 (1), 4581. 10.1038/s41467-021-24723-3 PubMed Abstract | 10.1038/s41467-021-24723-3 | Google Scholar 34321462 PMC8319183

[B148] Rasmussen M. Tan S. Somisetty V. S. Hutin D. Olafsen N. E. Moen A. (2021). PARP7 and Mono-ADP-Ribosylation Negatively Regulate Estrogen Receptor α Signaling in Human Breast Cancer Cells. Cells 10 (3). 623. 10.3390/cells10030623 PubMed Abstract | 10.3390/cells10030623 | Google Scholar 33799807 PMC8001409

[B149] Rodriguez K. M. Buch-Larsen S. C. Kirby I. T. Siordia I. R. Hutin D. Rasmussen M. (2021). Chemical Genetics and Proteome-wide Site Mapping Reveal Cysteine MARylation by PARP-7 on Immune-Relevant Protein Targets. Elife 10, e60480. 10.7554/eLife.60480 PubMed Abstract | 10.7554/eLife.60480 | Google Scholar 33475084 PMC7880690

[B150] Rosenthal F. Feijs K. L. H. Frugier E. Bonalli M. Forst A. H. Imhof R. (2013). Macrodomain-containing Proteins Are New Mono-ADP-Ribosylhydrolases. Nat. Struct. Mol. Biol. 20 (4), 502–507. 10.1038/nsmb.2521 PubMed Abstract | 10.1038/nsmb.2521 | Google Scholar 23474714

[B151] Roux K. J. Kim D. I. Burke B. (2013). BioID: a Screen for Protein-Protein Interactions. Curr. Protoc. Protein Sci. 74, 19–14. 10.1002/0471140864.ps1923s74 PubMed Abstract | 10.1002/0471140864.ps1923s74 | Google Scholar 24510646

[B152] Rulten S. L. Fisher A. E. O. Robert I. Zuma M. C. Rouleau M. Ju L. (2011). PARP-3 and APLF Function Together to Accelerate Nonhomologous End-Joining. Mol. Cel 41 (1), 33–45. 10.1016/j.molcel.2010.12.006 PubMed Abstract | 10.1016/j.molcel.2010.12.006 | Google Scholar 21211721

[B153] Ryu K. W. Nandu T. Kim J. Challa S. DeBerardinis R. J. Kraus W. L. (2018). Metabolic Regulation of Transcription through Compartmentalized NAD+ Biosynthesis. Science 360 (6389), eaan5780. 10.1126/science.aan5780 PubMed Abstract | 10.1126/science.aan5780 | Google Scholar 29748257 PMC6465534

[B154] Ryu K. W. Kim D.-S. Kraus W. L. (2015). New Facets in the Regulation of Gene Expression by ADP-Ribosylation and poly(ADP-Ribose) Polymerases. Chem. Rev. 115 (6), 2453–2481. 10.1021/cr5004248 PubMed Abstract | 10.1021/cr5004248 | Google Scholar 25575290 PMC4378458

[B155] Sanderson D. J. Cohen M. S. (2020). Mechanisms Governing PARP Expression, Localization, and Activity in Cells. Crit. Rev. Biochem. Mol. Biol. 55 (6), 541–554. 10.1080/10409238.2020.1818686 PubMed Abstract | 10.1080/10409238.2020.1818686 | Google Scholar 32962438

[B156] Schleicher E. M. Galvan A. M. Imamura-Kawasawa Y. Moldovan G.-L. Nicolae C. M. (2018). PARP10 Promotes Cellular Proliferation and Tumorigenesis by Alleviating Replication Stress. Nucleic Acids Res. 46 (17), 8908–8916. 10.1093/nar/gky658 PubMed Abstract | 10.1093/nar/gky658 | Google Scholar 30032250 PMC6158756

[B157] Schreiber V. Dantzer F. Ame J.-C. de Murcia G. (2006). Poly(ADP-ribose): Novel Functions for an Old Molecule. Nat. Rev. Mol. Cel Biol 7 (7), 517–528. 10.1038/nrm1963 PubMed Abstract | 10.1038/nrm1963 | Google Scholar 16829982

[B158] Seman M. Adriouch S. Haag F. Koch-Nolte F. (2004). Ecto-ADP-ribosyltransferases (ARTs): Emerging Actors in Cell Communication and Signaling. Cmc 11 (7), 857–872. 10.2174/0929867043455611 PubMed Abstract | 10.2174/0929867043455611 | Google Scholar 15078170

[B159] Sha H. Gan Y. Zou R. Wu J. Feng J. (2021). Research Advances in the Role of the Poly ADP Ribose Polymerase Family in Cancer. Front. Oncol. 11, 790967. 10.3389/fonc.2021.790967 PubMed Abstract | 10.3389/fonc.2021.790967 | Google Scholar 34976832 PMC8716401

[B160] Shacham T. Sharma N. Lederkremer G. Z. (2019). Protein Misfolding and ER Stress in Huntington's Disease. Front. Mol. Biosci. 6, 20. 10.3389/fmolb.2019.00020 PubMed Abstract | 10.3389/fmolb.2019.00020 | Google Scholar 31001537 PMC6456712

[B161] Shao C. Qiu Y. Liu J. Feng H. Shen S. Saiyin H. (2018). PARP12 (ARTD12) Suppresses Hepatocellular Carcinoma Metastasis through Interacting with FHL2 and Regulating its Stability. Cell Death Dis 9 (9), 856. 10.1038/s41419-018-0906-1 PubMed Abstract | 10.1038/s41419-018-0906-1 | Google Scholar 30154409 PMC6113207

[B162] Siva A. C. Raval-Fernandes S. Stephen A. G. LaFemina M. J. Scheper R. J. Kickhoefer V. A. (2001). Up-regulation of Vaults May Be Necessary but Not Sufficient for Multidrug Resistance. Int. J. Cancer 92 (2), 195–202. 10.1002/1097-0215(200102)9999:9999<::aid-ijc1168>3.0.co;2-7 PubMed Abstract | 10.1002/1097-0215(200102)9999:9999<::aid-ijc1168>3.0.co;2-7 | Google Scholar 11291045

[B163] Slade D. Dunstan M. S. Barkauskaite E. Weston R. Lafite P. Dixon N. (2011). The Structure and Catalytic Mechanism of a poly(ADP-Ribose) Glycohydrolase. Nature 477 (7366), 616–620. 10.1038/nature10404 PubMed Abstract | 10.1038/nature10404 | Google Scholar 21892188 PMC3184140

[B164] Sowa S. T. Galera-Prat A. Wazir S. Alanen H. I. Maksimainen M. M. Lehtiö L. (2021). A Molecular Toolbox for ADP-Ribosyl Binding Proteins. Cel Rep. Methods 1 (8), 100121. 10.1016/j.crmeth.2021.100121 10.1016/j.crmeth.2021.100121 | Google Scholar PMC8580838 34786571

[B165] Steffen J. D. Brody J. R. Armen R. S. Pascal J. M. (2013). Structural Implications for Selective Targeting of PARPs. Front. Oncol. 3, 301. 10.3389/fonc.2013.00301 PubMed Abstract | 10.3389/fonc.2013.00301 | Google Scholar 24392349 PMC3868897

[B166] Stevens L. A. Moss J. (2018). Mono-ADP-Ribosylation Catalyzed by Arginine-specific ADP-Ribosyltransferases. Methods Mol. Biol. 1813, 149–165. 10.1007/978-1-4939-8588-3_10 PubMed Abstract | 10.1007/978-1-4939-8588-3_10 | Google Scholar 30097866

[B167] Stiff T. O’Driscoll M. Rief N. Iwabuchi K. Löbrich M. Jeggo P. A. (2004). ATM and DNA-PK Function Redundantly to Phosphorylate H2AX after Exposure to Ionizing Radiation. Cancer Res. 64 (7), 2390–2396. 10.1158/0008-5472.can-03-3207 PubMed Abstract | 10.1158/0008-5472.can-03-3207 | Google Scholar 15059890

[B168] Stockinger B. Meglio P. D. Gialitakis M. Duarte J. H. (2014). The Aryl Hydrocarbon Receptor: Multitasking in the Immune System. Annu. Rev. Immunol. 32, 403–432. 10.1146/annurev-immunol-032713-120245 PubMed Abstract | 10.1146/annurev-immunol-032713-120245 | Google Scholar 24655296

[B169] Stubbs M. Veech R. L. Krebs H. A. (1972). Control of the Redox State of the Nicotinamide-Adenine Dinucleotide Couple in Rat Liver Cytoplasm. Biochem. J. 126 (1), 59–65. 10.1042/bj1260059 PubMed Abstract | 10.1042/bj1260059 | Google Scholar 4342386 PMC1178351

[B170] Sun Z. Brodsky J. L. (2019). Protein Quality Control in the Secretory Pathway. J. Cel Biol 218 (10), 3171–3187. 10.1083/jcb.201906047 PubMed Abstract | 10.1083/jcb.201906047 | Google Scholar PMC6781448 31537714

[B171] Suskiewicz M. J. Zobel F. Ogden T. E. H. Fontana P. Ariza A. Yang J.-C. (2020). HPF1 Completes the PARP Active Site for DNA Damage-Induced ADP-Ribosylation. Nature 579 (7800), 598–602. 10.1038/s41586-020-2013-6 PubMed Abstract | 10.1038/s41586-020-2013-6 | Google Scholar 32028527 PMC7104379

[B172] Takamura-Enya T. Watanabe M. Totsuka Y. Kanazawa T. Matsushima-Hibiya Y. Koyama K. (2001). Mono(ADP-ribosyl)ation of 2′-deoxyguanosine Residue in DNA by an Apoptosis-Inducing Protein, Pierisin-1, from Cabbage Butterfly. Proc. Natl. Acad. Sci. U.S.A. 98 (22), 12414–12419. 10.1073/pnas.221444598 PubMed Abstract | 10.1073/pnas.221444598 | Google Scholar 11592983 PMC60068

[B173] Tian L. Yao K. Liu K. Han B. Dong H. Zhao W. (2020). PLK1/NF-κB Feedforward Circuit Antagonizes the Mono-ADP-Ribosyltransferase Activity of PARP10 and Facilitates HCC Progression. Oncogene 39 (15), 3145–3162. 10.1038/s41388-020-1205-8 PubMed Abstract | 10.1038/s41388-020-1205-8 | Google Scholar 32060423

[B174] Timinszky G. Till S. Hassa P. O. Hothorn M. Kustatscher G. Nijmeijer B. (2009). A Macrodomain-Containing Histone Rearranges Chromatin upon Sensing PARP1 Activation. Nat. Struct. Mol. Biol. 16 (9), 923–929. 10.1038/nsmb.1664 PubMed Abstract | 10.1038/nsmb.1664 | Google Scholar 19680243

[B175] Todorova T. Bock F. J. Chang P. (2014). PARP13 Regulates Cellular mRNA post-transcriptionally and Functions as a Pro-apoptotic Factor by Destabilizing TRAILR4 Transcript. Nat. Commun. 5, 5362. 10.1038/ncomms6362 PubMed Abstract | 10.1038/ncomms6362 | Google Scholar 25382312 PMC4228382

[B176] Venkannagari H. Verheugd P. Koivunen J. Haikarainen T. Obaji E. Ashok Y. (2016). Small-Molecule Chemical Probe Rescues Cells from Mono-ADP-Ribosyltransferase ARTD10/PARP10-Induced Apoptosis and Sensitizes Cancer Cells to DNA Damage. Cel Chem. Biol. 23 (10), 1251–1260. 10.1016/j.chembiol.2016.08.012 PubMed Abstract | 10.1016/j.chembiol.2016.08.012 | Google Scholar 27667561

[B177] Verheugd P. Forst A. H. Milke L. Herzog N. Feijs K. L. H. Kremmer E. (2013). Regulation of NF-Κb Signalling by the Mono-ADP-Ribosyltransferase ARTD10. Nat. Commun. 4, 1683. 10.1038/ncomms2672 PubMed Abstract | 10.1038/ncomms2672 | Google Scholar 23575687

[B178] Vyas S. Chesarone-Cataldo M. Todorova T. Huang Y.-H. Chang P. (2013). A Systematic Analysis of the PARP Protein Family Identifies New Functions Critical for Cell Physiology. Nat. Commun. 4, 2240. 10.1038/ncomms3240 PubMed Abstract | 10.1038/ncomms3240 | Google Scholar 23917125 PMC3756671

[B179] Vyas S. Matic I. Uchima L. Rood J. Zaja R. Hay R. T. (2014). Family-wide Analysis of poly(ADP-Ribose) Polymerase Activity. Nat. Commun. 5, 4426. 10.1038/ncomms5426 PubMed Abstract | 10.1038/ncomms5426 | Google Scholar 25043379 PMC4123609

[B180] Wang C.-L. Tang Y. Li M. Xiao M. Li Q.-S. Yang L. (2021). Analysis of Mono-ADP-Ribosylation Levels in Human Colorectal Cancer. Cmar 13, 2401–2409. 10.2147/cmar.s303064 10.2147/cmar.s303064 | Google Scholar PMC7965690 33737837

[B181] Wang J. Zhu C. Song D. Xia R. Yu W. Dang Y. (2017). Epigallocatechin-3-gallate Enhances ER Stress-Induced Cancer Cell Apoptosis by Directly Targeting PARP16 Activity. Cell Death Discov. 3, 17034. 10.1038/cddiscovery.2017.34 PubMed Abstract | 10.1038/cddiscovery.2017.34 | Google Scholar 28698806 PMC5502302

[B182] Wang Z. Grosskurth S. E. Cheung T. Petteruti P. Zhang J. Wang X. (2018). Pharmacological Inhibition of PARP6 Triggers Multipolar Spindle Formation and Elicits Therapeutic Effects in Breast Cancer. Cancer Res. 78 (23), 6691–6702. 10.1158/0008-5472.can-18-1362 PubMed Abstract | 10.1158/0008-5472.can-18-1362 | Google Scholar 30297535

[B183] Wang Z. Michaud G. A. Cheng Z. Zhang Y. Hinds T. R. Fan E. (2012). Recognition of the Iso-ADP-Ribose Moiety in poly(ADP-Ribose) by WWE Domains Suggests a General Mechanism for poly(ADP-ribosyl)ation-dependent Ubiquitination. Genes Dev. 26 (3), 235–240. 10.1101/gad.182618.111 PubMed Abstract | 10.1101/gad.182618.111 | Google Scholar 22267412 PMC3278890

[B184] Weber P. C. Ohlendorf D. H. Wendoloski J. J. Salemme F. R. (1989). Structural Origins of High-Affinity Biotin Binding to Streptavidin. Science 243 (4887), 85–88. 10.1126/science.2911722 PubMed Abstract | 10.1126/science.2911722 | Google Scholar 2911722

[B185] Weixler L. Voorneveld J. Aydin G. Bolte T. M. H. R. Momoh J. Bütepage M. (2022). Systematic Analysis of ADP-Ribose Detection Reagents and Optimisation of Sample Preparation to Detect ADP-Ribosylation *In Vitro* and in Cells. bioRxiv. Preprint. 10.1101/2022.02.22.481411 10.1101/2022.02.22.481411 | Google Scholar

[B186] Weixler L. Schäringer K. Momoh J. Lüscher B. Feijs K. L. H. Žaja R. (2021). ADP-ribosylation of RNA and DNA: from *In Vitro* Characterization to *In Vivo* Function. Nucleic Acids Res. 49 (7), 3634–3650. 10.1093/nar/gkab136 PubMed Abstract | 10.1093/nar/gkab136 | Google Scholar 33693930 PMC8053099

[B187] Westcott N. P. Fernandez J. P. Molina H. Hang H. C. (2017). Chemical Proteomics Reveals ADP-Ribosylation of Small GTPases during Oxidative Stress. Nat. Chem. Biol. 13 (3), 302–308. 10.1038/nchembio.2280 PubMed Abstract | 10.1038/nchembio.2280 | Google Scholar 28092360 PMC5310985

[B188] Wigle T. J. Blackwell D. J. Schenkel L. B. Ren Y. Church W. D. Desai H. J. (2020). *In Vitro* and Cellular Probes to Study PARP Enzyme Target Engagement. Cel Chem. Biol. 27 (7), 877–887. 10.1016/j.chembiol.2020.06.009 PubMed Abstract | 10.1016/j.chembiol.2020.06.009 | Google Scholar 32679093

[B189] Williamson D. Lund P. Krebs H. (1967). The Redox State of Free Nicotinamide-Adenine Dinucleotide in the Cytoplasm and Mitochondria of Rat Liver. Biochem. J. 103 (2), 514–527. 10.1042/bj1030514 PubMed Abstract | 10.1042/bj1030514 | Google Scholar 4291787 PMC1270436

[B190] Wolozin B. Ivanov P. (2019). Stress Granules and Neurodegeneration. Nat. Rev. Neurosci. 20 (11), 649–666. 10.1038/s41583-019-0222-5 PubMed Abstract | 10.1038/s41583-019-0222-5 | Google Scholar 31582840 PMC6986315

[B191] Wu C. F. Xiao M. Wang Y. L. Threadgill M. D. Li M. Tang Y. (2020). PARP10 Influences the 1285 Proliferation of Colorectal Carcinoma Cells, a Preliminary Study. Mol. Biol. (Mosk) 54 (2), 252–261. 10.31857/s0026898420020184 PubMed Abstract | 10.31857/s0026898420020184 | Google Scholar 32392194

[B192] Yan F. Huang C. Wang X. Tan J. Cheng S. Wan M. (2020). Threonine ADP-Ribosylation of Ubiquitin by a Bacterial Effector Family Blocks Host Ubiquitination. Mol. Cel 78 (4), 641–652. 10.1016/j.molcel.2020.03.016 PubMed Abstract | 10.1016/j.molcel.2020.03.016 | Google Scholar 32330457

[B193] Yan Q. Xu R. Zhu L. Cheng X. Wang Z. Manis J. (2013). BAL1 and its Partner E3 Ligase, BBAP, Link Poly(ADP-Ribose) Activation, Ubiquitylation, and Double-Strand DNA Repair Independent of ATM, MDC1, and RNF8. Mol. Cel Biol 33 (4), 845–857. 10.1128/mcb.00990-12 PubMed Abstract | 10.1128/mcb.00990-12 | Google Scholar PMC3571337 23230272

[B194] Yang C.-S. Jividen K. Spencer A. Dworak N. Ni L. Oostdyk L. T. (2017). Ubiquitin Modification by the E3 Ligase/ADP-Ribosyltransferase Dtx3L/Parp9. Mol. Cel 66 (4), 503–516. 10.1016/j.molcel.2017.04.028 PubMed Abstract | 10.1016/j.molcel.2017.04.028 | Google Scholar PMC5556935 28525742

[B195] Yu Q. Wang B. Chen Z. Urabe G. Glover M. S. Shi X. (2017). Electron-Transfer/Higher-Energy Collision Dissociation (EThcD)-Enabled Intact Glycopeptide/Glycoproteome Characterization. J. Am. Soc. Mass. Spectrom. 28 (9), 1751–1764. 10.1007/s13361-017-1701-4 PubMed Abstract | 10.1007/s13361-017-1701-4 | Google Scholar 28695533 PMC5711575

[B196] Zafar M. K. Eoff R. L. (2017). Translesion DNA Synthesis in Cancer: Molecular Mechanisms and Therapeutic Opportunities. Chem. Res. Toxicol. 30 (11), 1942–1955. 10.1021/acs.chemrestox.7b00157 PubMed Abstract | 10.1021/acs.chemrestox.7b00157 | Google Scholar 28841374 PMC7135728

[B197] Žaja R. Aydin G. Lippok B. E. Feederle R. Lüscher B. Feijs K. L. H. (2020). Comparative Analysis of MACROD1, MACROD2 and TARG1 Expression, Localisation and Interactome. Sci. Rep. 10 (1), 8286. 10.1038/s41598-020-64623-y PubMed Abstract | 10.1038/s41598-020-64623-y | Google Scholar 32427867 PMC7237415

[B198] Zhang J. Snyder S. H. (1993). Purification of a Nitric Oxide-Stimulated ADP-Ribosylated Protein Using Biotinylated .beta.-nicotinamide Adenine Dinucleotide. Biochemistry 32 (9), 2228–2233. 10.1021/bi00060a014 PubMed Abstract | 10.1021/bi00060a014 | Google Scholar 8443164

[B199] Zhang Q. Piston D. W. Goodman R. H. (2002). Regulation of Corepressor Function by Nuclear NADH. Science 295 (5561), 1895–1897. 10.1126/science.1069300 PubMed Abstract | 10.1126/science.1069300 | Google Scholar 11847309

[B200] Zhang X.-N. Cheng Q. Chen J. Lam A. T. Lu Y. Dai Z. (2019). A Ribose-Functionalized NAD+ with Unexpected High Activity and Selectivity for Protein Poly-ADP-Ribosylation. Nat. Commun. 10 (1), 4196. 10.1038/s41467-019-12215-4 PubMed Abstract | 10.1038/s41467-019-12215-4 | Google Scholar 31519936 PMC6744458

[B201] Zhang Y. Liu S. Mickanin C. Feng Y. Charlat O. Michaud G. A. (2011). RNF146 Is a poly(ADP-Ribose)-Directed E3 Ligase that Regulates Axin Degradation and Wnt Signalling. Nat. Cel Biol 13 (5), 623–629. 10.1038/ncb2222 PubMed Abstract | 10.1038/ncb2222 | Google Scholar 21478859

[B202] Zhang Y. Wang J. Ding M. Yu Y. (2013). Site-specific Characterization of the Asp- and Glu-ADP-Ribosylated Proteome. Nat. Methods 10 (10), 981–984. 10.1038/nmeth.2603 PubMed Abstract | 10.1038/nmeth.2603 | Google Scholar 23955771

[B203] Zhao Y. Hu X. Wei L. Song D. Wang J. You L. (2018). PARP10 Suppresses Tumor Metastasis through Regulation of Aurora A Activity. Oncogene 37 (22), 2921–2935. 10.1038/s41388-018-0168-5 PubMed Abstract | 10.1038/s41388-018-0168-5 | Google Scholar 29515234

[B204] Zhao Y. Liang X. Wei L. Liu Y. Liu J. Feng H. (2021). RNF114 Suppresses Metastasis through Regulation of PARP10 in Cervical Cancer Cells. Cancer Commun. 41 (2), 187–191. 10.1002/cac2.12132 10.1002/cac2.12132 | Google Scholar PMC7896744 33417305

[B205] Zhen Y. Zhang Y. Yu Y. (2017). A Cell-line-specific Atlas of PARP-Mediated Protein Asp/Glu-ADP-Ribosylation in Breast Cancer. Cel Rep. 21 (8), 2326–2337. 10.1016/j.celrep.2017.10.106 PubMed Abstract | 10.1016/j.celrep.2017.10.106 | Google Scholar PMC5728415 29166620

